# Occupational health hazards of bidi workers and their families in India: a scoping review

**DOI:** 10.1136/bmjgh-2023-012413

**Published:** 2023-11-02

**Authors:** Jyoti Tyagi, Deepti Beri, Samiksha Ingale, Praveen Sinha, Soumyadeep Bhaumik

**Affiliations:** 1 Meta-Research and Evidence Synthesis, The George Institute for Global Health, New Delhi, Delhi, India; 2 Injury Division, The George Institute for Global Health, New Delhi, Delhi, India; 3 Non-Communicale Disease Division, WHO Country Office for India, New Delhi, Delhi, India

**Keywords:** Public Health, Health systems, Systematic review

## Abstract

**Background:**

Bidi workers and their families are exposed to harmful substances during bidi rolling, thereby jeopardising their health. We aimed to assess existing evidence on health conditions of bidi workers and their families in India.

**Methods:**

We searched nine databases and relevant websites, and conducted citation screening to identify primary studies assessing occupational health hazards of bidi workers and their families. Two authors independently conducted screening and data extraction. We synthesised the findings narratively in a structured fashion.

**Results:**

We found 3842 studies, out of which 95 studies met our eligibility criteria. High prevalence of disease conditions across all organ systems of the body was reported in bidi workers. Studies on female bidi workers showed decreased fertility (n=2), increased frequency of miscarriages (n=1) and higher risk of cervical cancer (n=1). Pregnant bidi workers were at an increased risk of anaemia and pregnancy-induced hypertension (n=2), higher frequency of neonatal deaths (n=1), stillbirths (n=1) and premature births (n=1) in comparison with non-bidi workers. Babies born to bidi workers reported low birth weight (n=5). Evidence from cohort studies suggests causal nature of the exposure to the disease condition.

**Conclusion:**

Our review shows that bidi rolling leads to numerous occupational health hazards in bidi workers and their family members. It is essential to provide alternative livelihoods, and safe and protective working environment, and cover bidi workers under various social security provisions to alleviate the deleterious effect of bidi making at home. It is also important to shift bidi making away from home and strengthen existing regulations and promulgation of new provisions, including India’s Occupational Safety, Health, and Working Conditions Code 2020.

**WHAT IS ALREADY KNOWN ON THIS TOPIC**

- The bidi industry employs 4.9 million workers in India. Women and children comprise 90% of the workforce, as apparently their nimble fingers can roll bidis better.
- Bidi making is a labour-intensive process, most of which is unorganised and is done from home of bidi workers. They are exposed to harmful substances like nicotine, tar, dust and other particles (through cutaneous and nasopharyngeal route), thus potentially leading to occupational health hazards.

**WHAT THIS STUDY ADDS**

- To the best of our knowledge, this is the first evidence synthesis concerning occupational health hazards in bidi workers and their families. Ninety-five studies from India reported high prevalence of various diseases among bidi workers.
- Female bidi workers who constitute majority of the workforce have a high proportion of and are at greater risk of gynaecological disorders.
- Newborns and children of bidi workers are at higher risk of suffering from low birth weight and stunted growth.

**HOW THIS STUDY MIGHT AFFECT RESEARCH, PRACTICE OR POLICY**

- In view of the scientific evidence on the health hazards of bidi work, policymakers in India may consider classifying bidi rolling as a hazardous process under various forms of domestic legislation and policies at the national and subnational levels including India’s Occupational Safety, Health, and Working Conditions Code 2020. This will facilitate shifting of bidi rolling from home to factories and thus bidi workers can be covered by more formal protections, including better wages.
- There is also a need to develop and implement initiatives for providing alternative livelihoods and safe working conditions for bidi workers so that detrimental effects of bidi rolling are mitigated.
- There is potential correlation indicated from case–control studies and causal relationship from limited cohort studies. Our review recommends need for future research by conducting more long-term studies to establish temporality, along with interventional trials especially designed for women and children.

## Introduction

Tobacco kills more than 8 million people globally and leads to 229.77 million disability-adjusted life years lost annually.^1 2^ Out of these, India accounts for 1.35 million deaths.^3^ Unlike other countries, cigarette is not the most common tobacco product consumed in India. Bidi, an indigenous smoking tobacco product, made by tobacco flakes rolled in tendu leaves and tied with a thread, is the most smoked product with 85% market share.^4^ Most of the bidi rolling work is done by women from homes, continuously exposing them to nicotine, tar, unburnt tobacco dust and other toxic particles that pass through cutaneous and pharyngeal route.^5^ A study by Gupta *et al* (Bombay cohort study) conducted in Mumbai, India reported the relative risk for all-cause mortality among bidi smokers was 64% higher as compared with never users of tobacco.^6^

It is also known that bidi workers are exposed to several occupational health hazards because of long working hours and unhealthy work environment.^7^ Occupational health risk in bidi workers, their families and communities is an important cause of concern.^8–10^ The bidi industry is estimated to employ about 4.9 million people and supports nearly 2.2 million people from the tribal communities, who are engaged in plucking and sale of tendu leaves, predominantly in the unorganised sector.^5 11^ Due to this unorganised nature of bidi work, there is lack of awareness on using safety measures such as wearing protective gears like masks and gloves, and washing hands, which leads to prolonged exposure to tobacco dust in bidi workers.^12^

While many primary studies exist, there is no evidence synthesis on occupational health hazards due to tobacco exposure during bidi making in bidi workers and their families from India. Thus, we aimed to fill this gap by conducting a scoping review on the topic, in view of the Occupational Safety, Health, and Working Conditions Code 2020 (OSH Code), gazetted in 2020 in India, which does not explicitly classify bidi work as a ‘hazardous process’^13^ and the Bidi Workers Welfare Act of 1976 (an act to provide financing measures to promote the welfare of persons engaged in bidi establishments) being repealed in 2019.^14^ As we aimed to map and report broad but detailed available evidence on health hazards of bidi rolling, we undertook the scoping review approach.

The deliberations from the results of the study can inform policy options for the union and the state Governments of India (GoI), to improve health and well-being of bidi workers and their families and facilitate their shifting to alternative livelihood. This will also fulfil India’s commitment towards Articles 17 and 18 (welfare of tobacco growers and workers including their health) of the World Health Organization’s Framework Convention on Tobacco Control (WHO FCTC), to which India is a prominent signatory.^15^

## Methods

The review is reported in accordance with the Preferred Reporting Items for Systematic review and Meta-Analyses extension for Scoping Reviews (PRISMA-ScR) 2020 guidelines and checklist which is presented in online supplemental appendix 1. The protocol was registered a priori in medRxiv^16^ (https://www.medrxiv.org/content/10.1101/2022.03.24.22272764v1).

10.1136/bmjgh-2023-012413.supp1 Supplementary data

### Eligibility criteria

We included studies meeting the following criteria:

- Population/problem: on bidi workers (any role, function or type of enterprise) and their families in the working environment.
- Interest area: on any of the following:
  - Occupational health hazards of bidi workers.
  - Health risks/hazards in families of bidi workers.
- Setting: the study should be conducted in India.
- Study design: any study providing primary data (qualitative or quantitative), irrespective of study design or peer review status.

We included original research articles in the English language. We did not use any time limit. We did not include animal or laboratory studies on effects of constituents of bidi.

### Information sources

#### Electronic database search

We searched the following nine electronic databases from date of inception:

1. PubMed.
2. EMBASE+EMBASE Classic.
3. CINAHL (Cumulative Index to Nursing and Allied Health Literature).
4. Environment complete–EBSCO.
5. GreenFILE–EBSCO.
6. Web of Science.
7. WHO-IRIS (Institutional Repository for Information Sharing).
8. WHO Global Index Medicus.
9. Archives of Indian Labour (https://indianlabourarchives.org/).

All search strategies are presented in online supplemental appendix 2.

10.1136/bmjgh-2023-012413.supp2 Supplementary data

#### Searching other resources

We additionally identified potential studies from the grey literature using the same search terms used for electronic database search. We also contacted experts in the field, did citation screening of included studies and hand searched relevant websites. We searched websites of the Ministry of Health and Family Welfare (MoHFW) and Ministry of Labour and Employment of the GoI. We also searched other labour organisations, trade unions and non-profit organisations working on tobacco control or welfare of bidi workers in India. The complete list is presented in online supplemental appendix 3.

10.1136/bmjgh-2023-012413.supp3 Supplementary data

### Selection of sources of evidence

Three review authors (JT, DB, SI) independently screened the titles and/or abstracts from electronic database search using Rayyan (a web-based application).^17^ This was followed by full-text evaluation, as per inclusion criteria by at least two review authors (JT, SI). Similarly, grey literature was screened by title and/or abstract and then by full-text evaluation, for possible inclusion by two review authors (JT, SI). Any discrepancies were resolved by a consensus with the other review author (SB).

### Data charting process and content analysis

Three review authors (JT, DB, SI) conducted data extraction supported by SB. We used a standardised data extraction form to collect the data on the following information: study design, country, setting, sample size, income or wages, type and nature of worker engagement, type of enterprise and disease conditions.

### Synthesis of results

We synthesised the results narratively with a description of summary of results from primary studies, without conducting any additional quantitative analysis. We synthesised and reported the results in a structured manner, categorising the occupational health hazards in bidi workers and their families. We reported findings from special population of bidi workers, separately. This special population included children involved in bidi work, pregnant and lactating bidi workers.

### Patient and public involvement

We did not involve patients and the public in this study.

## Results

### Study selection

**Figure 1:**
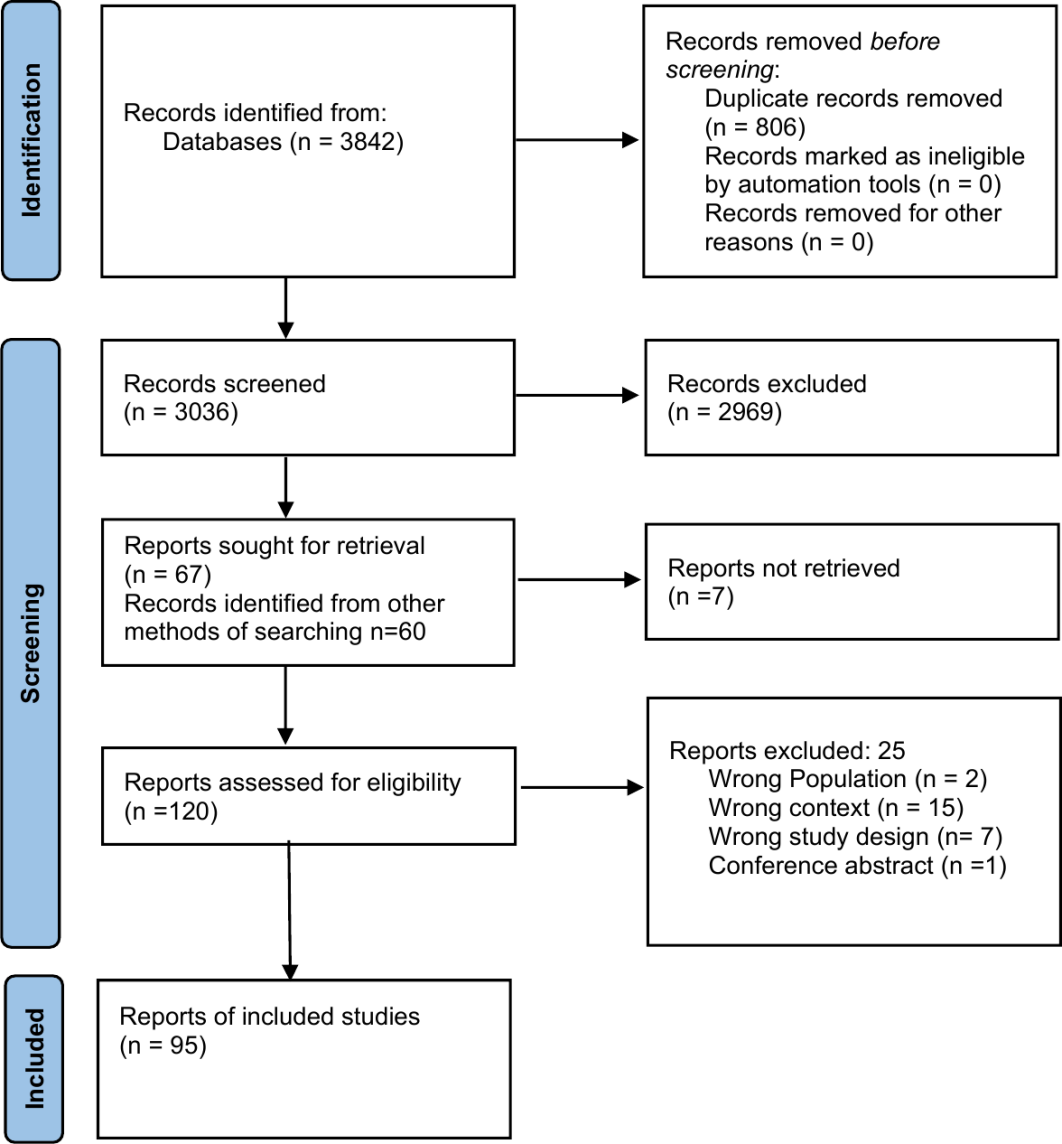
Preferred Reporting Items for Systematic Reviews and Meta-Analyses flow chart showing selection of studies.

We retrieved 3842 records through electronic database searching. After removing duplicates and screening 3036 records based on title and/or abstract, we included 67 studies for full-text review. We additionally identified 60 records through citation screening and grey literature search for the full-text review. Finally, we included 95 studies (96 reports) in the review that met the inclusion criteria. The list of studies excluded at the full-text level, along with reasons, is presented in online supplemental appendix 4. The PRISMA flow chart showing study selection is shown in figure 1.

10.1136/bmjgh-2023-012413.supp4 Supplementary data

### Characteristics of included studies

Overall, we found 95 studies (96 reports) assessing occupational health hazards of bidi workers and their families.^8–10 12 18–108^ We found eight studies from the grey literature, while 87 studies were published. Majority of these studies were cross-sectional (n=58), followed by case–control (n=26), cohort (n=3), mixed-methods (n=3), case study (n=1), quasi experimental study (n=1) and qualitative studies (n=4). Many studies were conducted in Karnataka (n=22), Tamil Nadu (n=22), West Bengal (n=14) and Maharashtra (n=12). The state-wise distribution of studies is presented in online supplemental appendix 5. An interactive version of this distribution is also available at: https://public.flourish.studio/visualisation/10184632/. The characteristics of the included studies are summarised in online supplemental appendix 6.

10.1136/bmjgh-2023-012413.supp5 Supplementary data

10.1136/bmjgh-2023-012413.supp6 Supplementary data

We found 87 studies assessing occupational health hazards in bidi workers.^8–10 12 18–43 45 47–51 53–67 69 71–84 87–90 92–108^ We synthesised evidence on domains based on the medical specialty, to enable presentation which could be well understood by clinicians and policymakers, as well as by the general public. Table 1 depicts the summary of the number of included studies and their epidemiological designs presented according to the distribution of disease conditions.

**Table 1:** Summary characteristics of included study

Sr no	Disease condition	Number of studies	Study design
**Occupational health hazards in bidi workers**			
1.	Respiratory diseases	44	Cross-sectional: 36 Case–control: 7 Quasi-experimental: 1
2.	Musculoskeletal diseases	41	Cross-sectional: 35 Case–control: 4 Mixed-methods: 1 Case study: 1
3.	Gastrointestinal diseases	30	Cross-sectional: 26 Case–control: 3 Case study: 1
4.	Neurological diseases	13	Cross-sectional: 11 Case–control: 2
5.	Gynaecological diseases	13	Cross-sectional: 11 Case–control: 2
6.	Anaemia and malnutrition	9	Cross-sectional: 8 Case–control: 1
7.	Mental health conditions	6	Cross-sectional: 6
8.	Dermatological conditions	27	Cross-sectional: 21 Case–control: 4 Mixed-methods: 2
9.	Ophthalmological disorders	32	Cross-sectional: 28 Case–control: 4
10.	Genitourinary tract disorders	3	Cross-sectional: 2 Case–control: 1
11.	Endocrine disorders	7	Cross-sectional: 6 Case–control: 1
12.	Oral health conditions	5	Cross-sectional: 5
13.	Oncological conditions	5	Cross-sectional: 4 Case–control: 1
14.	Haematological disorders	4	Case–control: 4
15.	Otolaryngology diseases	14	Cross-sectional: 14
16.	Cardiovascular diseases	15	Cross-sectional: 9 Case–control: 6
17.	Genotoxicity	15	Cross-sectional: 3 Case–control: 12
**Occupational health hazard(s) in special populations of bidi workers**			
Pregnant bidi workers			
1.	Anaemia and nutritional deficiencies	1	Cohort study: 1
2.	Gynaecological problems	3	Case–control: 1 Cohort: 2
3.	Genotoxicity	2	Case–control: 1 Cohort: 1
4.	Lactating bidi workers	No studies found	
5.	Child labourers in bidi rolling	2	Cross-sectional: 2
**Occupational health hazard(s) in families of bidi workers**			
1.	Disorders of newborns	7	Cross-sectional: 1 Case–control: 4 Cohort: 2
2.	Respiratory disorders	3	Cross-sectional: 1 Cohort: 1 Mixed-methods: 1
3.	Gastrointestinal disorders	1	Cohort: 1
4.	Nutritional deficiencies	2	Cross-sectional: 1 Cohort: 1

**Figure 2:**
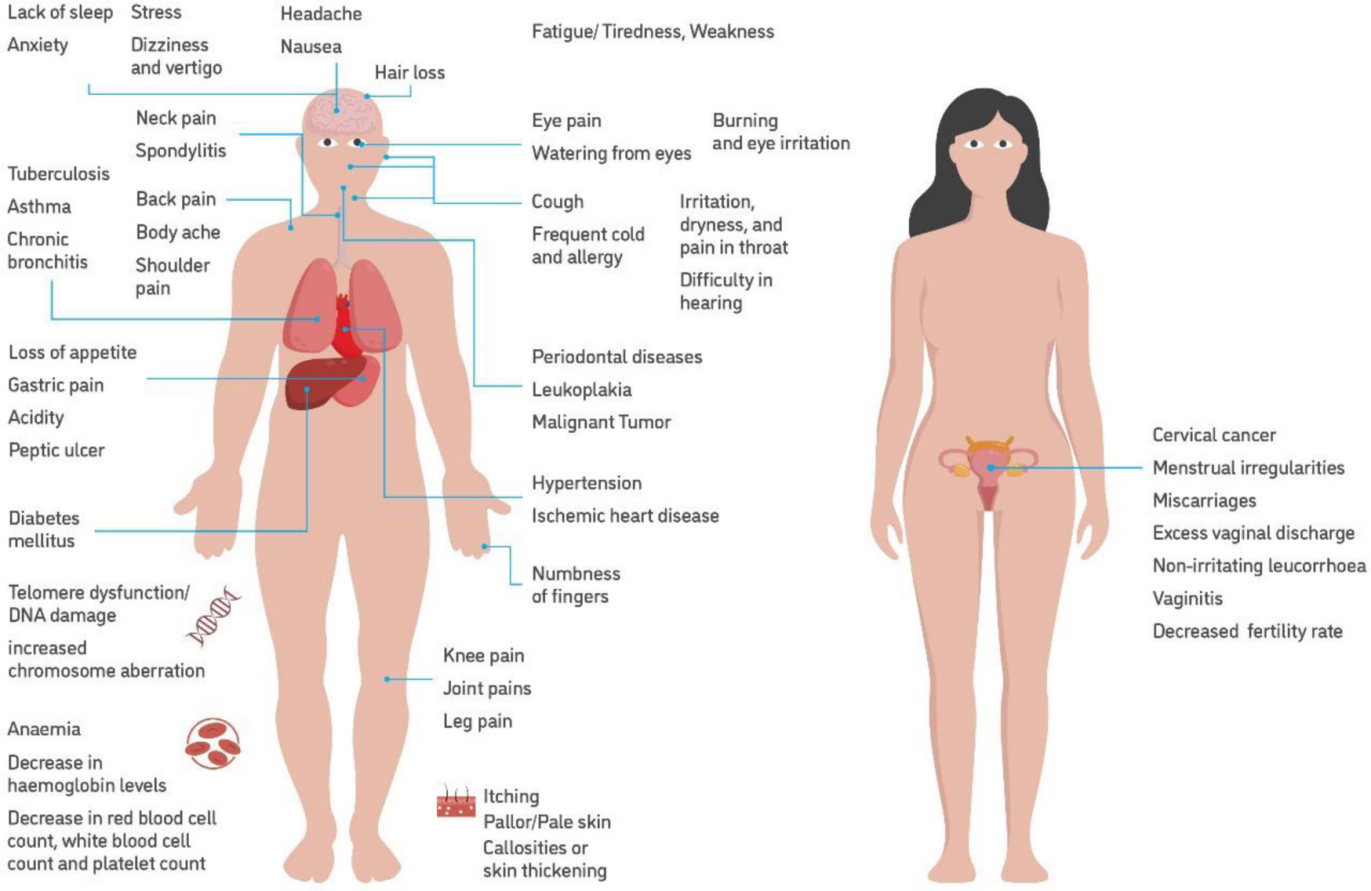
Occupational health hazards of bidi workers.

**Figure 3:**
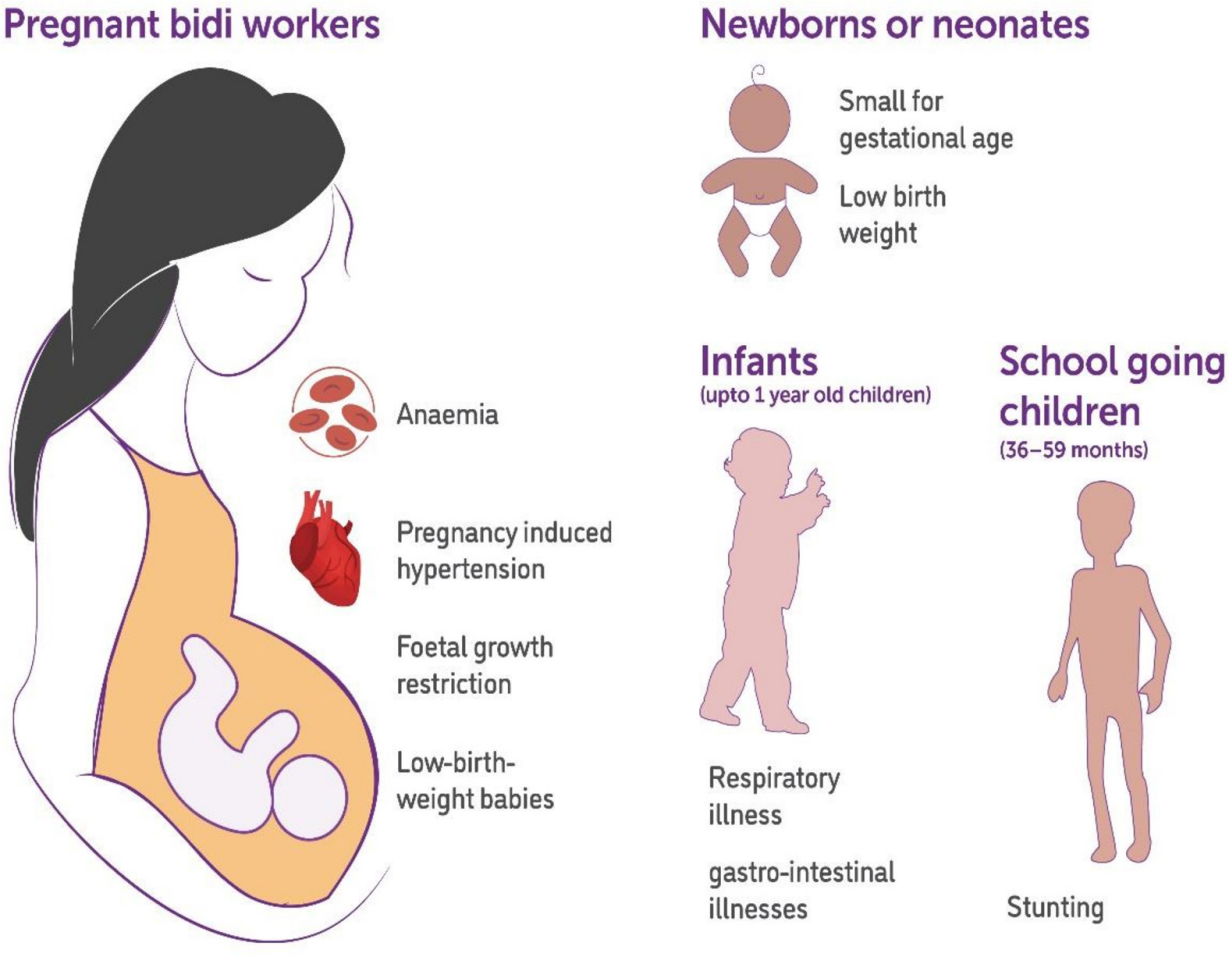
Occupational health hazards in pregnant bidi workers and children.

Figure 2 depicts the distribution of diseases in bidi workers, while figure 3 depicts distribution of diseases in pregnant bidi workers and their children.

### Occupational health hazards in bidi workers

#### Respiratory diseases

We found 44 studies on respiratory diseases in bidi workers,^8–10 12 18 19 21 25 26 32 34 36 40 49 50 56 59 60 62 63 65 66 69 71–73 75 77–82 84 90 97 98 100 102–107^ reporting high prevalence of tuberculosis, asthma and chronic bronchitis. Out of these, 36 were cross-sectional studies,^8–10 12 18 19 21 26 34 40 56 59 60 62 63 65 66 69 71–73 77–82 90 97 98 100 102–107^ seven case–control studies^25 32 36 50 75 84 107^ and one quasi-experimental study.^49^ The prevalence of respiratory disorders reported in cross-sectional studies of bidi workers varied from 6.6% to 52.5%.^19 21 40 59 60 62 65 73 77–79 81 100 103 104^

Five studies reported higher prevalence of any respiratory disease (6.09–10.3% vs 1.0–7.25%),^25 75 84^ tuberculosis (6.6% vs 0%)^107^ and asthma (9.1–25% vs 3.3–12%)^50 107^ in female bidi workers when compared with non-bidi workers. The case–control study evaluating pulmonary health reported a statistically significant higher forced expiratory flow and peak expiratory flow rate among non-bidi workers, in comparison with bidi workers.^32^ Another case–control study assessing pulmonary functional status reported various respiratory impairments (restrictive, obstructive and ‘combined restrictive and obstructive’) which were higher among bidi workers (23.53%) than non-bidi workers (3.56%).^36^

The quasi-experimental study evaluated the effect of providing a simple protective mask to bidi workers and reported a significant decrease in the prevalence of respiratory symptoms due to the intervention. However, there was no significant improvement reported between forced expiratory volume in 1st sec, FEF_25_ (forced mid-expiratory flow) and FEF_75_.^49^

#### Musculoskeletal diseases

We found 41 studies on musculoskeletal diseases in bidi workers.^8–10 12 18 19 21 22 25 33 34 40 47 50 53 56 58 59 62 63 65 66 71–73 77–82 84 87 92 98 100 102–104 106 107^ Out of these, 35 were cross-sectional studies,^8–10 12 18 19 21 22 33 34 40 53 56 58 59 63 65 66 71–73 77–82 87 92 98 100 102–104 106^ four case–control studies,^25 50 84 107^ one mixed-methods study^87^ and one used a case study design.^47^

Studies reported that prevalence of any musculoskeletal symptom in bidi workers was as high as 34.6% up to 87.0%.^18 21 59 62 66 81 100 104^ Studies reported various symptoms of musculoskeletal diseases like back pain, body ache and shoulder pain. The case–control studies^25 50 84 107^ compared female bidi workers with non-bidi workers and consistently found higher prevalence of musculoskeletal diseases (34.8% vs 8%).^84^

#### Gastrointestinal diseases

We found 30 studies on gastrointestinal diseases in bidi workers.^8 9 12 18 19 21 22 25 27 40 45 47 53 56 59 64 65 72 73 77–79 81 82 102–107^ There were 26 cross-sectional studies,^8 9 12 18 19 21 22 27 40 53 56 59 64 65 72 73 77–79 81 82 102–106^ three case–control studies^25 45 107^ and one study that used a case study approach.^47^

The prevalence of any gastrointestinal symptom ranged from 3.9% to 70% in 11 studies.^9 18 21 22 40 56 59 65 78 79 104^ Studies reported several gastrointestinal symptoms with most studies reporting very high prevalence of loss of appetite, peptic ulcer and gastric pain.^8 9 27 47 59 72 73 77 81 82 102 105^ The case–control studies reported higher prevalence of gastrointestinal disorders (26.96% vs 10%), stomach pain (39.69 vs 15%) and gas (71.1% vs 32.8%) in female bidi workers in comparison with non-bidi workers.^25 107^ The case study also reported stomach-related pains including cramps, gas, as well as spasmodic pains leading to diarrhoea in female bidi workers.^47^

#### Neurological diseases

We found 13 studies on neurological disorders in bidi workers.^22 25 27 40 59 62 71 72 77 79 100 104 107^ Eleven studies were cross-sectional^22 27 40 59 62 71 72 77 79 100 104^ and two were case–control studies.^25 107^

Cross-sectional studies reported prevalence of several neurological symptoms like numbness of fingers, dizziness and vertigo. The case–control studies reported female bidi workers having neurological symptoms (9.57% vs 1.5%) and giddiness (36.6% vs 5.01%) as compared with non-bidi workers.^25 107^

#### Gynaecological diseases

We found 13 studies reporting gynaecological disorders among female bidi workers.^8 9 21 25 37 40 53 62 64 71 73 100 107^ Out of these, 11 were cross-sectional^8 9 21 37 40 53 62 64 71 73 100^ and 2 were case–control studies.^25 107^

A high prevalence of overall gynaecological diseases was noted in female bidi workers, ranging from 2% to 42%.^37 40 62 64 71 100^ Studies reported prevalence of several gynaecological symptoms like menstrual irregularities and disturbances, excess vaginal discharge and non-irritating leucorrhoea. Findings from case–control studies indicated adverse reproductive outcomes in female bidi workers, with 37.1% of bidi workers with no tobacco smoking/chewing habits experiencing miscarriages in comparison with 11.5% in non-bidi workers.^107^ Another case–control study also noted an increased frequency of abortion (12.16% vs 2.42%), uterine problems, (17.5% vs 6%) and a decreased fertility rate (84% vs 91.94%) in bidi workers as compared with controls who were not bidi workers.^25^

#### Anaemia and malnutrition

We found nine studies reporting anaemia and other nutritional disorders in bidi workers.^9 21 25 27 53 60 78 103 105^ Eight of them were cross-sectional studies^9 21 27 53 60 78 103 105^ and one was a case–control study.^25^ Studies reported prevalence of anaemia among bidi workers, ranging from 6% to 33.3%,^9 21 53 60 78 103 105^ while 13.5% of bidi workers were obese.^27^ The case–control study reported anaemia in female bidi workers (41.00% vs 9.5%) as compared with the controls.^25^

#### Mental health conditions

We found six cross-sectional studies related to mental health conditions of bidi workers.^19 34 59 81 103 104^ Studies reported high prevalence of lack of sleep (ranging from 5.4% to 12.73%)^34 59 81 104^ and anxiety (from 3.64% to 8.8%).^34 103^

#### Dermatological conditions

We found 27 studies reporting dermatological conditions in bidi workers.^8–10 12 18 25 27 38 56 59–61 66 71–73 78 82–84 87 98–100 102–104^ Twenty-one studies were cross-sectional,^8–10 12 18 27 38 56 59 60 66 71–73 78 82 98 100 102–104^ four were case–control^25 61 84 99^ and two used mixed-methods design.^83 87^ Overall, studies reported prevalence of skin conditions like itching, pallor, callosities and tanning of skin that varied from 3% to 56.7% among bidi workers. The case–control studies consistently reported higher prevalence of callosities over fingers and feet (56.04% vs 0.3%; p<0.05), localised nail changes, (24.18% vs 0%; p<0.001)^61^ and hair loss (86.33% vs 69%)^25^ in bidi workers in comparison with non-bidi workers.

#### Ophthalmological disorders

We found 32 studies reporting ophthalmological disorders in bidi workers.^8 10 19 21 22 25 27 34 38 40 50 55 59 60 62 63 65 66 71 77 78 80–82 90 98–100 102 104 106 107^ There were 28 cross-sectional^8 10 19 21 22 27 34 38 40 55 59 60 62 63 65 66 71 77 78 80–82 90 98 100 102 104 106^ and four case–control studies.^25 50 99 107^

Studies reported prevalence of ophthalmological conditions like burning, itching, redness of eye, eye watering and irritation, and diminished, defective or blurred vision, ranging from 7.3% to 81%, in bidi workers.^19 21 40 59 60 62 66 77 78 81 106 107^ The case–control studies reported female bidi workers with higher prevalence of any eye symptom (50.33–66.5% vs 36.1–41%) and eye watering (22.8% vs 9.8%) as compared with non-bidi workers.^25 50 99 107^

#### Genitourinary tract disorders

We found three studies reporting genitourinary tract disorders in bidi workers.^10 25 73^ One was a case–control study^25^ and other two were cross-sectional studies.^10 73^ Creatinine value (g/24 hours) of female bidi workers was significantly higher in comparison with the control subjects (mean and SD=4.56±2.94 vs 1.40±0.32), representing kidney dysfunction.^10^

#### Endocrine disorders

We found seven studies which reported endocrine disorders in bidi workers.^25 56 58–60 66 97^ There were six cross-sectional studies^56 58–60 66 97^ and one case–control study.^25^

Studies reported prevalence of 3.4–17% of diabetes mellitus and 1.67% of hypothyroidism in female bidi workers.^56 58–60 66 97^ The case–control study reported that 3.04% female bidi workers had diabetes mellitus in comparison with only 1% of matched controls of non-bidi workers.^25^

#### Oral health conditions

We found five cross-sectional studies on oral health of bidi workers.^28 66 73 95 108^ Studies reported on prevalence of several oral health conditions like periodontal diseases, leucoplakia and oral submucous fibrosis in bidi workers, ranging from 3.1% to 67.2%. One study reported a statistically significant association between working experiences of bidi rolling and occurrence of oral premalignant lesions in bidi workers (leucoplakia=34–45 years, 27.6% vs 16–25 years, 1.6%; p=0.001).^108^

#### Oncological conditions

We found five studies on cancer in bidi workers.^9 19 51 73 97^ Out of these, four were cross-sectional^9 19 73 97^ studies while one was a case–control study.^51^ Findings from cross-sectional studies reported bidi workers suffering from lung carcinoma (2%) and unspecified cancer (2–3.3%). The case–control study reported that female workers engaged in bidi rolling were two times more likely to suffer from cervical cancer as compared with non-bidi workers (adjusted (adjusted for effects of tobacco chewing among participants) OR (95% CI)=1.913 (1.215, 3.01); p=0.005).^51^

#### Haematological disorders

We found four case–control studies reporting deranged haematological parameters in bidi workers.^22 25 41 107^ These studies reported significant decrease in mean haemoglobin levels (g/L) and platelet count (10^9^/L) in female bidi workers as compared with non-bidi workers (p<0.001).

#### Otolaryngology diseases

We found 14 studies on otolaryngology diseases in bidi workers.^8 9 18 19 27 34 38 72 73 80 82 102 104 106^ All were cross-sectional studies. These studies reported high prevalence of several ear, nose and throat symptoms like cough, frequent cold, irritation, dryness and pain in the throat in bidi workers.

#### Cardiovascular diseases

We found 15 studies on cardiovascular diseases in bidi workers.^22 25 27 41 50 58–60 66 73 76 81 97 99 107^ There were nine cross-sectional studies^27 58–60 66 73 76 81 97^ and six case–control studies.^22 25 41 50 99 107^

Studies reported high prevalence of hypertension, hypotension and ischaemic heart diseases in bidi workers. One study observed electrocardiogram (ECG) changes in female bidi workers and reported no changes in ECG parameter. The mean QTc interval was within the normal range of 400–440 ms, while and mean Tp-e interval was 79.47±3.2 ms.^76^

Studies reported significant increase in serum total cholesterol (mean and SD=180.74±20.78 vs 161.36±16.91) and triglycerides (mean and SD=152±27.8 vs 113.20±10.19) in exposed cases as compared with non-bidi worker controls (p<0.001).^22 41^

#### Genotoxicity

We found 15 studies assessing genotoxicity among bidi workers.^20 23 24 29–31 42 43 48 54 57 67 94 99 101^ There were 12 case–control studies^23 24 29–31 43 48 54 57 67 99 101^ and three cross-sectional studies.^20 42 94^ These studies typically measured several biological markers related to toxicity (for example, urinary thioether, and salivary and urinary cotinine levels) or studied genetic aberrations (for example, measurement of telomere and mutagenicity).

Five studies assessing urinary thioether levels in workers engaged in bidi rolling and tobacco processing activities^30 31 43 48 94^ reported significantly high mean urinary thioether levels in bidi workers than those in controls.

Five studies evaluated cotinine levels in bidi workers and tobacco processors.^23 24 30 31 48^ The mean urinary cotinine levels in tobacco processors with a history of tobacco habits were significantly higher than the no-tobacco control group (3.46±0.95 and 3.57±0.46 vs 1.80±0.58 mM/M creatinine; p<0.02) indicating high levels of tobacco dust exposure.^24^

Five studies evaluated chromosomal aberration in bidi workers and tobacco processors^29 43 57 67 99^ and reported significant increase in chromosome aberration in bidi workers exposed to tobacco dust.

Three studies assessed telomere length in bidi workers exposed to tobacco dust^43 54 57^ and found significant increase in the comet and tail length of telomeres in bidi workers occupationally exposed to tobacco dust.

Two studies assessed urinary mutagenicity due to tobacco exposure in bidi workers with no tobacco habit and tobacco processors (no tobacco and masheri habit), respectively.^24 31^ Results showed that exposure to tobacco during bidi rolling resulted in an increased mutagenicity in TA98 in absence of metabolic activation, but no mutagenic activity was detected in TA100. This implies genotoxic hazard of occupational exposure to tobacco.^31^

Detailed results on genotoxicity are reported in online supplemental appendix 7.

10.1136/bmjgh-2023-012413.supp7 Supplementary data

### Results of syntheses of qualitative and mixed-methods studies in bidi workers

The analysis of qualitative and mixed-methods studies led to the following general themes being derived:

#### Health hazards and working conditions in bidi workers

All the studies captured health problems in bidi workers. Bidi workers reported dizziness, fever, chest pain, and pain in the hands, legs, shoulders, neck and back due to prolonged working hours in the same posture for bidi rolling. Many participants reported burning of eyes, respiratory irritation, chronic bronchitis, tuberculosis and overall health deterioration because of prolonged tobacco dust inhalation.^44 70 74 83 87 88 91^ Female bidi workers who rolled bidis at home in small huts with inadequate ventilation reported that tobacco dust remains in the home where their families and they spend most of their time, leading to respiratory illnesses.^83^

#### Occupational health hazard(s) in special populations of bidi workers

We defined three special populations a priori for synthesis and disaggregated results in relation to pregnant and lactating women and children involved in bidi work.

##### Pregnant bidi workers

We found six studies assessing the impact of bidi rolling on pregnant bidi workers.^35 46 68 85 89 93^

###### Anaemia and nutritional deficiencies

One cohort study assessed anaemia in pregnant women engaged in bidi rolling^35^ and reported a statistically significant higher proportion of anaemia in pregnant bidi workers when compared with non-bidi workers (OR=1.4; p=0.037). 35

###### Gynaecological problems

Three studies reported gynaecological-related disorders.^35 89 93^ Two were cohort studies^35 93^ while one was a case–control study.^89^ The case–control study was conducted^89^ among women admitted in labour ward and reported a high fetoplacental ratio in the bidi worker group as compared with the non-bidi worker group (fetoplacental ratio±SD (0.2029±0.028 vs 0.1798±0.017); p<0.001). A high fetoplacental ratio denotes foetal hypoxia.

Both the cohort studies reported 3.5 times higher risk of having pregnancy-induced hypertension and 2.7 times higher risk of fetal growth restriction in bidi workers as compared with non-bidi workers.^35 93^

###### Genotoxicity

Two studies estimated the urine cotinine levels in pregnant bidi workers (one case–control and one cohort study).^89 93^ The biomarker denotes exposure to harmful components of tobacco which impacts foetal circulation and subsequently results in negative foetal outcomes. Both the studies reported a significantly higher amount of urine cotinine levels in pregnant bidi workers, ranging from 2 ng/mL to 500 ng/mL. The cohort study reported a high serum cotinine value in both maternal (125 ng/mL) and cord blood (110 ng/mL) samples of the exposed group.^93^

##### Lactating bidi workers

We did not find any study assessing occupational health hazards in lactating bidi workers.

##### Child labourers in bidi rolling

We found two cross-sectional studies assessing occupational health hazards in child labourers involved in bidi rolling.^39 96^ These studies reported high prevalence of headache, body pain, asthma, respiratory inflammations, sinusitis and allergic rhinitis in children.

### Results of syntheses of qualitative and mixed-methods studies in special populations of bidi workers

Female bidi workers reported that their babies often fall sick with vomiting, diarrhoea and fever. These women reported lack of appetite and prolonged fatigue, with no energy to even speak. They also experienced frequent abortions and gave birth to stillborn or Low BirthWeight (LBW) babies.^70 74^ Numerous young girls who were involved in bidi rolling reported irregular periods which they were not willing to discuss with their family members.^70^

#### Occupational health hazard(s) in families of bidi workers

We found 13 studies assessing occupational health hazards in the families and communities of bidi workers.^25 35 46 52 68 73 74 79 83 85 86 89 93^

##### Disorders of newborns

Seven studies reported disorders in newborns of women who were engaged in bidi work during pregnancy.^25 35 68 73 85 89 93^ Out of these, one was a cross-sectional study,^73^ four were case–control studies^25 68 85 89^ and two were cohort studies.^35 93^

The case–control studies^68 89^ reported that the mean birth weight of babies born to mothers engaged in bidi rolling during pregnancy was lower than babies of non-bidi rolling mothers. Mothers working in bidi rolling establishment reported a significant 310 g reduction in birth weight (mean and SD=2.350±0.420 kg vs 2.660±0.350 kg; p<0.001) of their babies.^89^ Another case–control study reported that occupational tobacco exposure in pregnant bidi workers led to a significant reduction in cord serum leptin (adjusted (adjusted for maternal gestational hypertension, prematurity and birth weight) mean difference (95% CI)=−4.5 ng/mL (−8.82, –0.19); p=0.041). Reduced cord serum leptin is a marker of alteration of neuroendocrine function of the fetus and indicates an increased risk of small for gestational age (SGA) neonates.^85^

The hospital-based prospective cohort study^93^ found a statistically significant decrease in adjusted (adjusted for age, parity, body mass index, weight gain, anaemia, hypertension, gestational diabetes mellitus, gestation week, bidi rolling) mean difference of birth weight (−104 g) of babies and 1.75 times higher risk for SGA babies in pregnant women involved in bidi rolling process during pregnancy, and at least 1 year before pregnancy.

Another cohort study^35^ found a statistically significant increased risk of LBW neonates (OR=1.9; p=0.001) among bidi worker mothers as compared with non-bidi worker mothers.

##### Respiratory disorders

Three studies reported on respiratory disorders in families and communities of bidi workers. There was one cohort, one cross-sectional and one mixed-methods study.^46 52 83^ A study conducted on a birth cohort living in urban slums reported infants belonging to households involved in bidi rolling activity were at 1.3 times more risk of suffering from respiratory illness as compared with households with no bidi rolling activity (adjusted (adjusted for birth weight, number of siblings, socioeconomic status, household size, religion, type of family and presence of animals in the house) rate ratio (RR) (95% CI)=1.3 (1.1, 1.5); p=0.001).^46^

##### Gastrointestinal disorders

A cohort study conducted on a birth cohort in an urban slum reported a 1.3 times increased risk of gastrointestinal illnesses in infants born in households where bidi rolling activity was undertaken, as compared with households with no bidi rolling activity (adjusted (adjusted for birth weight, number of siblings, duration of exclusive breast feeding, socioeconomic status, household size, religion, type of family and presence of animals in the house) RR (95% CI)=1.3 (1.1, 1.5); p=0.003).^46^

##### Nutritional deficiencies

A cross-sectional study conducted on preschool children (36–59 months) reported higher odds of stunting in children whose mothers were bidi workers (adjusted (adjusted for child’s age, child’s sex, birth order, birth interval, LBW, duration of breast feeding, mother’s age at birth, mother’s occupation and child attending Anganwadi centre) OR (95% CI)=1.92 (1.18 to 3.12); p<0.010).^86^ Another cohort study reported increased risk of overall morbidity (defined as any infectious morbidity including gastrointestinal illnesses, respiratory illnesses, bronchitis, pneumonia) and non-infectious morbidity (injuries, anaemia, congenital diseases, convulsions, neonatal jaundice, birth-related morbidities, loss of appetite and malnutrition)) in infants living in households engaged in bidi rolling activity (adjusted RR (95% CI)=1.2 (1.1, 1.4); p<0.001).^46^

## Discussion

Our study reports evidence from 95 studies across 14 Indian states on health hazards in bidi workers and their families, showing very high prevalence of respiratory (up to 52.5%), musculoskeletal (up to 87%), gastrointestinal (up to 70%), neurological (up to 60%), skin (up to 37%) and other conditions across the organ system.

We also identified that female bidi workers constituted majority of the workforce, and in them, studies reported higher risk of having cervical cancer and chromosomal aberrations. Pregnant female bidi workers were at increased risk of having pregnancy-induced hypertension and reported high amount of urine cotinine levels. Infants and children, whose mothers or any other family member were engaged in bidi rolling activities, were at higher risk of having respiratory and gastrointestinal illnesses and stunted growth.

Similar findings have been reported in other studies including informal domestic workers and subcontracted piece-rate home workers such as in textiles, matchbox making, firecrackers and waste pickers facing significant health perils like bidi workers.^42 109^

Evidence from our study should be read in cognisance with what is already known about the economic and working conditions of bidi workers, including the monograph developed by the MoHFW, GoI, earlier in 2008.^5^ Bidi workers usually do not have proper housing, they need to sit in the same position for long hours and segregation of their workplace and dwelling is not feasible. The bidi industry constitutes mostly of women who are exploited by getting less wages than their counterparts in the manufacturing sector.^7^

Although bidi rolling began in the factory setting (in early 20th century), over the last three decades, bidi manufacturers have increasingly shifted bidi work from factories to households.^110^ Currently, most of the bidi manufacturing is done through Own Account Manufacturing Enterprises run in private dwellings of bidi workers. This shift (from factory to household) was attributed to strict government rules, regulations and policies enforced on the organised sector and the tax liberalisation in the unorganised sector.^7^

With 99.31% of bidi workers working from home and not covered under important regulations in India for their welfare, their physical environment such as facilities of creche and shelter to rest is found to be poor.^7 111^ Bidi workers are known to be underpaid, with their wages not increasing substantially even though profits of the bidi industry have increased.^7^ The report of the Committee on Subordinate Legislation (16th Lok Sabha) noted that ‘bidi workers are the most exploited among all rural labour, and women workers are most affected. They are paid much below the National Minimum Wage Norms and are unable to meet even their basic needs.’^112^ Given the poor socioeconomic status of bidi workers, it is their right to get sociolegal protection under a lot of labour welfare legislation. They should be entitled to social security measures mandated by the Minimum Wages Act 1948 and the Provident Funds Act 1925, for fixing, reviewing, and revising minimum wages and receiving post-retirement benefits for the employees or their legal heirs, in case of death of the employee, respectively. In addition to this, bidi workers and their families should be supported to seek medical benefits, education, housing and health insurance.

Findings from the qualitative and mixed-methods studies align with outcomes of quantitative studies, with persistently reporting occupational diseases across various organ systems of bidi workers and their families. Where evidence from case–control studies (n=26) existed, it consistently and uniformly showed that risk or prevalence of various health conditions was significantly higher in bidi workers, in comparison with non-bidi workers. This indicated correlation between risk factor and the diseases, whereas cohort studies (n=3) indicated a potential causal relationship between exposure and the disease. However, there is a dearth of cohort studies to confirm this causal relationship, with no clinical trials for many disease conditions known to be associated with bidi work. Hence, we recommend the Indian Council of Medical Research conduct a research priority setting exercise and develop a national task force project to undertake multistate longitudinal studies and cluster randomised controlled trials on interventions, especially designed for women and children, to reduce risk of diseases associated with bidi rolling. This can enable assessment of causality, as well as build an evidence base to inform strategic decisions for improving health of bidi workers and their families.

Our study, overall, reports multitude of health conditions which bidi workers and their families face. This implies the need for governmental regulations in the domain that are favourable for improvement of health and well-being of these workers and their families. Developing a large-scale plan for imparting additional skills for alternative sources of livelihood, which are equally more remunerative and healthier, is urgently required. The ‘Healthy Option’ special project for promotion of alternative occupations through skill development of bidi workers and their dependents under Pradhan Mantri Kaushal Vikas Yojana (2016–2020) needs to be expanded and upscaled.^113^

In light of evidence from multiple studies from India, policymakers might consider reviewing the OSH Code 2020^13^ as well as instituting other appropriate legislation, strategies and programmes such that the health and welfare of bidi workers are protected. Such policies should ensure that bidi workers and their families have unrestricted access to health, welfare and alternative livelihood schemes, which remains unaddressed. Additional incentives like linking them to Mahatma Gandhi National Rural Employment Guarantee Act 2005 for assured wages, in lieu of bidi rolling, must also be explored. These policies will strengthen India’s commitment towards Articles 17 and 18 of the WHO FCTC, which relate to the ‘provision of support for economically viable alternative activities’ and ‘protection of the environment and the health of persons’, respectively.^114^

The strength of our study lies in the use of comprehensive search strategy across multiple databases and grey literature search. We acknowledge that internal studies conducted by the bidi industry or any research commissioned by public authorities not available publicly might have been missed. Nevertheless, we used standard evidence synthesis practices with screening, and data extraction being performed independently by at least two review authors and cross-checked by a third review author.

## Conclusion

Bidi work leads to deleterious health hazards in bidi workers who are majorly women and their families. Provisions for improving the current working conditions of home-based bidi workers can be made through regulatory changes viz. the classification of bidi work as a hazardous process under the OSH Code 2020 and/or other suitable regulations. Long-term changes in their working conditions by disincentivising bidi rolling process and shifting bidi rollers to alternative livelihoods through skill development training programmes are suggested.

## Data Availability

Data are available upon reasonable request. Data are available upon reasonable request to the corresponding author.
