# Supplementary material for: Occupational health hazards of bidi workers and their families in India: a scoping review
Source: BMJ Glob Health. 2023 Nov 2;8(11):e012413. doi: 10.1136/bmjgh-2023-012413 (PMC10626877; doi:10.1136/bmjgh-2023-012413)
Supplement: Supplementary data [file bmjgh-2023-012413supp002.pdf]

Appendix 2: Search strategies

Pubmed

No.	Search terms	Number of hits
#1	"bidi" OR bidis OR "beedi" OR beedis	193,25
#2	India[Mesh] OR India* OR Andaman OR Nicobar OR Andhra OR Arunachal OR Assam OR Bihar OR Chandigarh OR Chhattisgarh OR "Dadra and Nagar Haveli" OR Daman OR Diu OR Delhi OR Goa OR Gujarat OR Haryana OR Himachal OR Jammu OR Kashmir OR Jharkhand OR Karnataka OR Kerala OR Lakshadweep OR "Madhya Pradesh" OR Maharashtra OR Manipur OR Meghalaya OR Mizoram OR Nagaland OR Orissa OR Odisha OR Pondicherry OR Punjab OR Rajasthan OR Sikkim OR "Tamil Nadu" OR Telangana OR Tripura OR "Uttar Pradesh" OR Uttarakhand OR "Bengal"	963,760
#3	#1 AND #2	**1859**

EMBASE ovid

No.	Search terms	Number of hits
#1	(bidi OR bidis OR beedi OR beedis).mp	902
#2	exp India/ OR (India* OR Andaman OR Nicobar OR Andhra OR Arunachal OR Assam OR Bihar OR Chandigarh OR Chhattisgarh OR "Dadra and Nagar Haveli" OR Daman OR Diu OR Delhi OR Goa OR Gujarat OR Haryana OR Himachal OR Jammu OR Kashmir OR Jharkhand OR Karnataka OR Kerala OR Lakshadweep OR "Madhya Pradesh" OR Maharashtra OR Manipur OR Meghalaya OR Mizoram OR Nagaland OR Orissa OR Odisha OR Pondicherry OR Punjab OR Rajasthan OR Sikkim OR "Tamil Nadu" OR Telangana OR Tripura OR "Uttar Pradesh" OR Uttarakhand OR Bengal).mp	385,643
#3	#1 AND #2	**504**
#4	Limit to pubmed-not-medline	**0**

CINAHL

No.	Search terms	Number of hits
#1	bidi OR bidis OR beedi OR beedis	208
#2	(MH India+) OR India* OR Andaman OR Nicobar OR Andhra OR Arunachal OR Assam OR Bihar OR Chandigarh OR Chhattisgarh OR "Dadra and Nagar Haveli" OR Daman OR Diu OR Delhi OR Goa OR Gujarat OR Haryana OR Himachal OR Jammu OR Kashmir OR Jharkhand OR Karnataka OR Kerala OR Lakshadweep OR "Madhya Pradesh" OR Maharashtra OR Manipur OR Meghalaya OR Mizoram OR Nagaland OR Orissa OR Odisha OR Pondicherry OR Punjab OR Rajasthan OR Sikkim OR "Tamil Nadu" OR Telangana OR Tripura OR "Uttar Pradesh" OR Uttarakhand OR Bengal	235,776
#3	#1 AND #2	**125**

Environment complete-EBSCO

No.	Search terms	Number of hits
#1	TX “bidi” OR bidis OR “beedi” OR beedis	616
#2	SU India OR TX India* OR Andaman OR Nicobar OR Andhra OR Arunachal OR Assam OR Bihar OR Chandigarh OR Chhattisgarh OR “Dadra and Nagar Haveli” OR Daman OR Diu OR Delhi OR Goa OR Gujarat OR Haryana OR Himachal OR Jammu OR Kashmir OR Jharkhand OR Karnataka OR Kerala OR Lakshadweep OR “Madhya Pradesh” OR Maharashtra OR Manipur OR Meghalaya OR Mizoram OR Nagaland OR Orissa OR Odisha OR Pondicherry OR Punjab OR Rajasthan OR Sikkim OR “Tamil Nadu” OR Telangana OR Tripura OR “Uttar Pradesh” OR Uttarakhand OR “Bengal”	395,390
#3	#1 AND #2	**258**

GreenFILE – EBSCO

No.	Search terms	Number of hits
#1	TX “bidi” OR bidis OR “beedi” OR beedis	27
#2	SU India OR TX India* OR Andaman OR Nicobar OR Andhra OR Arunachal OR Assam OR Bihar OR Chandigarh OR Chhattisgarh OR “Dadra and Nagar Haveli” OR Daman OR Diu OR Delhi OR Goa OR Gujarat OR Haryana OR Himachal OR Jammu OR Kashmir OR Jharkhand OR Karnataka OR Kerala OR Lakshadweep OR “Madhya Pradesh” OR Maharashtra OR Manipur OR Meghalaya OR Mizoram OR Nagaland OR Orissa OR Odisha OR Pondicherry OR Punjab OR Rajasthan OR Sikkim OR “Tamil Nadu” OR Telangana OR Tripura OR “Uttar Pradesh” OR Uttarakhand OR “Bengal”	522,16
#3	#1 AND #2	**13**

Web of Science

No.	Search terms	Number of hits
#1	bidi OR bidis OR beedi OR beedis	736
#2	ALL=India OR India* OR Andaman OR Nicobar OR Andhra OR Arunachal OR Assam OR Bihar OR Chandigarh OR Chhattisgarh OR "Dadra and Nagar Haveli" OR Daman OR Diu OR Delhi OR Goa OR Gujarat OR Haryana OR Himachal OR Jammu OR Kashmir OR Jharkhand OR Karnataka OR Kerala OR Lakshadweep OR "Madhya Pradesh" OR Maharashtra OR Manipur OR Meghalaya OR Mizoram OR Nagaland OR Orissa OR Odisha OR Pondicherry OR Punjab OR Rajasthan OR Sikkim OR "Tamil Nadu" OR Telangana OR Tripura OR "Uttar Pradesh" OR Uttarakhand OR Bengal	305,5373
#3	#1 AND #2	**420**

WHO-IRIS

No.	Search terms	Number of hits
#1	Bidi	565 only 500 downloadable
#2	beedi	45
	Total	545
	After duplicate removal	525

WHO Global Index Medicus

No.	Search terms	Number of hits
#1	"bidi" OR bidis OR "beedi" OR beedis	611
#2	India	41997
#3	#1 AND #2	**93**

Archives of Indian Labour

No.	Search terms	Number of hits
#1	"bidi"	118
#2	"beedi"	20
#3	#1 AND #2	**138**
