# Supplementary material for: Occupational health hazards of bidi workers and their families in India: a scoping review
Source: BMJ Glob Health. 2023 Nov 2;8(11):e012413. doi: 10.1136/bmjgh-2023-012413 (PMC10626877; doi:10.1136/bmjgh-2023-012413)
Supplement: Supplementary data [file bmjgh-2023-012413supp003.pdf]

Appendix 3 List of organisations

1.	International labour organization https://www.ilo.org/Search5/search.do?sitelang=en&locale=en_EN&consumercode=ILOHQ_STELLENT_PUBLIC&searchWhat=bidi&searchLanguage=en
2.	AF Development Care (AFDC) https://afdc.in/listing_detail.php?programme=17 Knowledge Gap in Existing Research on India's Women Beedi Rollers & Alternative Livelihood Option
3.	Voluntary Health Association of India https://vhai.org/
4.	Center for Workers' Management (CWM)
5.	Centre for health and social justice (CHSJ)
6.	Public health foundation of India
7.	National Institute of Health and Family Welfare
8.	Campaign for Tobacco-Free Kids www.tobaccofreecenter.org
9.	PATH Canada http://citeseerx.ist.psu.edu/viewdoc/download?doi=10.1.1.613.2222&rep=rep1&type=pdf
