# Supplementary material for: Occupational health hazards of bidi workers and their families in India: a scoping review
Source: BMJ Glob Health. 2023 Nov 2;8(11):e012413. doi: 10.1136/bmjgh-2023-012413 (PMC10626877; doi:10.1136/bmjgh-2023-012413)
Supplement: Supplementary data [file bmjgh-2023-012413supp004.pdf]

Appendix 4 Reasons for exclusion at full text level

No.	Citation	Reason for exclusion
1.	Ali MS, Kumar P. Responding to the needs of beedi workers after the outbreak of the COVID-19 pandemic. Social Work with Groups. 2021;44(4):390-6.	Wrong context no details on occupational health of beedi rollers
2.	Aluckal E. Association between sleep deprivation and chronic periodontitis among beedi workers of Mangalore city. JOURNAL OF SLEEP RESEARCH. 2015; 25:134-.	Wrong context not on occupational diseases and a conference abstract
3.	Bhatia R, Sharma VK. Occupational dermatoses: An Asian perspective. Indian J Dermatol Venereol Leprol. 2017;83(5):525-35.	Wrong study design- literature review
4.	Chatterjee M. Occupational health of self-employed women workers. Experiences from community based studies of the Self-Employed Women's Association (SEWA). Health Millions. 1993;1(1):13-7.	Wrong study design- pooled results from studies
5.	Cheriamane D. Beedi rolling-its impact on workers health. European Respiratory Journal. 2014;44.	Conference abstract and full study included
6.	Dharmalingam A. FEMALE BEEDI WORKERS IN A SOUTH INDIAN VILLAGE. ECONOMIC AND POLITICAL WEEKLY. 1993;28(27):1461-8.	Wrong study design- article from economic and political weekly no primary data
7.	Dharmalingam A. The social context of family size preferences and fertility behaviour in a south Indian village. Genus. 1996;52(1):83-103.	Wrong context. The study does not provide details of occupational or environmental hazard
8.	Dharmalingam A, Morgan SP. Women's Work, Autonomy, and Birth Control: Evidence from Two South Indian Villages. Population Studies. 1996;50(2):187-201.	Wrong context. The study does not provide details of occupational or environmental hazard
9.	Gulati GM, Isaac TMT, Klein WA. When a workers' cooperative works: The case of Kerala Dinesh Beedi. UCLA LAW REVIEW. 2002;49(5):1417-54.	Wrong context. The study does not provide details of occupational or environmental hazard
10.	Jacob AM, Kathyayani BV. A Study to Assess the Effectiveness of Yogic Postures on Back Pain among Beedi Rolling Women in a Selected Rural Area at Mangalore. International Journal of Nursing Education. 2017;9(2):1-5.	Wrong context. The study does not provide details of occupational or environmental hazard
11.	Jeyaseelan L, Rao PS. Effect of occupation on menstrual cycle length: causal model. Hum Biol. 1995;67(2):283-90.	Wrong study design
12.	Jha S. Household-specific variables and	Wrong context. The study does not

	forest dependency in an Indian hotspot of biodiversity: challenges for sustainable livelihoods. Environment, Development & Sustainability. 2009;11(6):1215-23.	provide details of occupational or environmental hazard
13.	Karunanithi G. Plight of pledged children in beedi works. ECONOMIC AND POLITICAL WEEKLY. 1998;33(9):450-2.	Wrong study design - editorial
14.	Kumar P, Kamath A, Nayak R, Kulkarni MM, Kamath VG, Mullapudi S, et al. Beedi Rollers' Perception toward Alternative Means of Livelihood amid Economic Benefits. Indian J Community Med. 2021;46(2):268-72.	Wrong context. The study does not provide details of occupational or environmental hazard
15.	Lando HA, Kabir Z, Mohan P, Panneer S. Analysis of the effect of bidi regulations and its influence on tobacco control in India. Tobacco Induced Diseases. 2018;16:221.	Wrong context. The study does not provide details of occupational or environmental hazard
16.	Mallick J, Satpathy S. Estimation of Women Beedi Workers in India and Their Socio-economic Condition. INDIAN JOURNAL OF LABOUR ECONOMICS. 2021;64(2):499-521.	Wrong context. The study does not provide details of occupational or environmental hazard
17.	Mohandas M, Kumar PV. IMPACT OF CO-OPERATIVISATION ON WORKING-CONDITIONS - STUDY OF BEEDI INDUSTRY IN KERALA. ECONOMIC AND POLITICAL WEEKLY. 1992;27(26):1333-8.	Wrong study design
18.	Rayhan S, Banerjee A. Measuring the child's home learning environment and its associated factors in Malda: A micro-level study in India. CHILDREN AND YOUTH SERVICES REVIEW. 2021;125.	Wrong study population
19.	Rout SK, Balu RK, Selvaraj S. Health and livelihood paradox: Understanding livelihood and poverty situation of tobacco workers. Respiratory Medicine. 2013;107:S12.	Wrong context and a Conference abstract
20.	Tom J, Francis SS. Occupational health risks of beedi rollers reinvestigated: Issues and evidence. International Journal of Pharma Medicine and Biological Sciences. 2013;2(4):28-33.	Wrong context. The study does not provide details of occupational or environmental hazard
21.	Kumar Arun GRK. Age at menarche and menopause among Bidi workers women of Sagar district of Central India. Voice of Intellectual Man- An International Journal 2014;4(2):91-6.	Wrong context. The study does not provide details of occupational or environmental hazard
22.	Supase A OK, Mukherjee K, Singh K, Chaturvedi P. Alternative livelihood for bidi workers: a study based on primary research on home-based bidi rollers of Solapur city	Wrong context. The study does not provide details of occupational or environmental hazard

	of Maharashtra. International Journal of Community Medicine and Public Health. 2020;7(11):17-24.	
23.	Bagwe AN, and R.A. Bhisey Mutagenicity of processed bidi tobacco: possible relevance to bidi industry workers. Mutation Res. 1991;261:93-9	Wrong population. The study does not provide details of occupational or environmental hazard
24.	Purkait SKaBKS. Occupational Health Hazards of Women Beedi Workers in Rural India. International Journal of Science, Engineering and Technology Research. 2015;4(5).	Wrong study design- reviewed literature and pooled results from studies
25.	Kumar Arun GRK. Natural Selection among Bidi Workers of Sagar (MP), India. Asian Man (The) – An International Journal. 2015; 9(1):79-82	Wrong context. The study does not provide details of occupational or environmental hazard
