# Supplementary material for: Occupational health hazards of bidi workers and their families in India: a scoping review
Source: BMJ Glob Health. 2023 Nov 2;8(11):e012413. doi: 10.1136/bmjgh-2023-012413 (PMC10626877; doi:10.1136/bmjgh-2023-012413)
Supplement: Supplementary data [file bmjgh-2023-012413supp005.pdf]

Appendix 5: Map showing state-wise distribution of studies on occupational health of bidi workers from across India

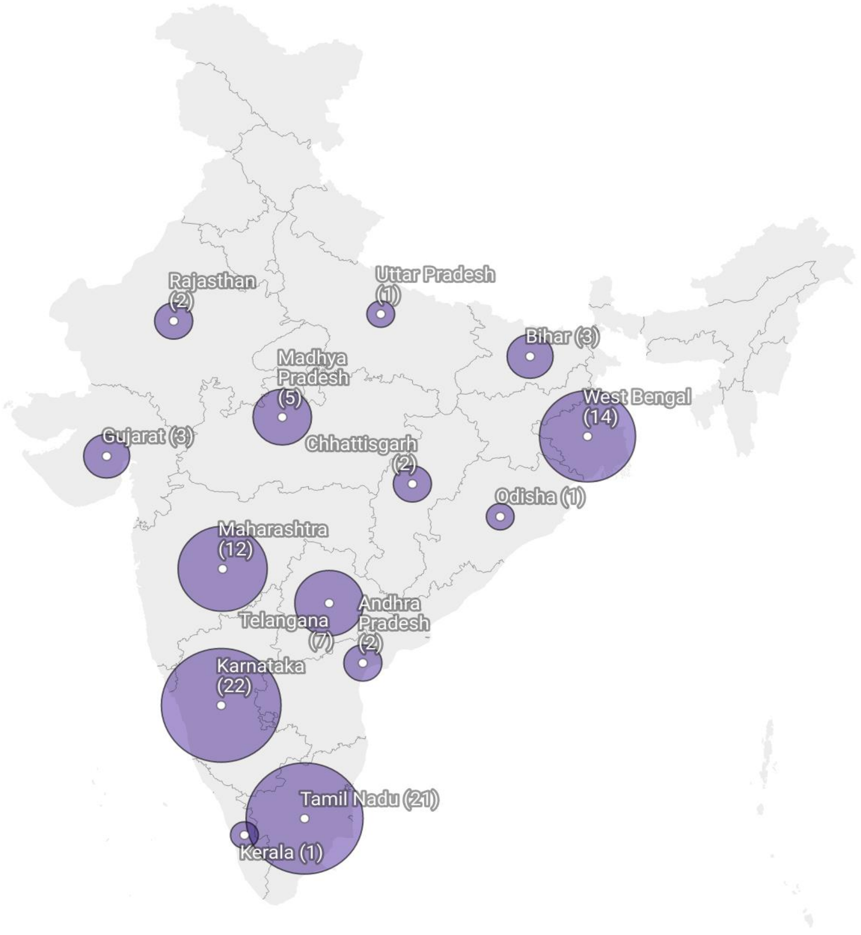

Map: MRES Unit, The George Institute of Global Health, India • Map data: © OSM • Created with Datawrapper
