# Supplementary material for: Occupational health hazards of bidi workers and their families in India: a scoping review
Source: BMJ Glob Health. 2023 Nov 2;8(11):e012413. doi: 10.1136/bmjgh-2023-012413 (PMC10626877; doi:10.1136/bmjgh-2023-012413)
Supplement: Supplementary data [file bmjgh-2023-012413supp006.pdf]

Appendix 6: Characteristics of included studies table

Sr no.	Citation	Title	Study design	Name of the State	Setting	Population	Sample size	Income/Wages	Disease categories	Funding
1	Adhikary 2016	A Study of Water Crisis and Occupational Health Hazards among Bidi workers of Purulia, West Bengal	Cross-sectional study	West Bengal	Households	Households employed in bidi rolling occupation were selected;	663 Bidi workers (124 households)	Mean annual family level income is only Rs. 15500/- and annual mean health expenditure of BPL households is Rs. 5480/- only with a standard deviation of Rs. 2179/- & Rs. 1238/- respectively.	Musculoskeletal diseases; Gastrointestinal diseases; Skin problems; Otolaryngology (ENT) diseases; respiratory diseases	Not reported
2	Alamelu mangai 2020	A survey on health status of women bidi workers in Mukkudal	Cross-sectional study	Tamil Nadu	Mukkudal Town panchayat Tirunelveli district under Tamil Nadu state	Bidi workers residing in Mukkudal Town panchayat Tirunelveli district	400 Bidi workers	Not reported	Oncological conditions; Musculoskeletal diseases; Ophthalmologic al disorders; Otolaryngology (ENT) diseases; respiratory diseases; Neurological	Not reported

									problems; Gastro-intestinal problems. Mental health conditions	
3	Ancy 2021	Comparative Evaluation of Salivary Sialic Acid Levels Among Bidi Rollers and Tobacco Users in Mangalore, South India	Cross-sectional study	Karnataka	Dental screening and treatment camps in rural and urban areas in Mangalore, conducted by the Department of Public Health Dentistry, Yenepoya Dental College and patients who visited the Department of Oral Medicine and Radiology of	Subjects aged between 30 and 60 years who consented to participate in the study were grouped as: Group 1. Individuals who have been bidi rollers for at least 10 years, who do not smoke or use smokeless tobacco, and who are not exposed to second hand smoke at home or at work.	35 Bidi rollers, 35 Tobacco smokers, 35 Smokeless tobacco users,35 Subjects with no tobacco exposure	Not reported	Genotoxicity	Nil

					Yenepoya Dental College	Group 2: - Individuals who have smoked tobacco for at least ten years and do not use smokeless tobacco. Group 3: Individuals who have used smokeless tobacco for at least 10 years without smoking tobacco. Group 4: Individuals who had not used tobacco for at least 30 days before the examination.				
4	Anil 2012	Study of morbidity pattern of	Cross-sectional study	Karnataka	Community based – Urban area	Bidi workers in the urban field practice	113 Bidi workers	Not reported	Musculoskeletal; Gynaecological diseases;	Not reported

		female bidi workers in the urban field practice area of Mangalore, Southern India				area of Mangalore			Ophthalmological disorders; Gastrointestinal ; Respiratory; Anaemia and nutritional deficiency	
5	Annalakshmi 2013	Health assessment in women bidi rollers in melapalayam, tirunelveli	Cross-sectional study	Tamil Nadu	Melapalaym of Tirunelveli District	Women bidi workers from the town panchayat of Mukkudal	1000 Bidi workers	Not reported	Musculoskeletal diseases; Gastro-intestinal problems; neurological problems; Ophthalmological disorders	Not reported
6	Annalakshmi 2013a	Health assessment in women bidi rollers in melapalayam, tirunelveli – Routine Haematological analysis of selected women bidi rollers of Melapalayam	Case control study	Tamil Nadu	Melapalayam of Tirunelveli District	Bidi workers residing in Melapalayam of Tirunelveli District	150 Bidi workers and 150 Control subjects	Not reported	Haematological disorders; Lipid profile	Not reported
7	Bagwe 1993	Occupational exposure to tobacco and resultant genotoxicity in	Case control study	Maharashtra	Not reported	Only women tobacco processing plant workers	105 Bidi workers. tobacco processing plant	Not reported	Genotoxicity	Not reported

		bidi industry workers				(TPPW)and bidi rollers	workers (TPPW): 67 Control subjects: 91			
8	Bagwe 1995	Occupational exposure to unburnt bidi tobacco elevates mutagenic burden among tobacco processors	Case control study	Southern India	Tobacco processing plant	Women tobacco processors working in a small town in Southern India along with controls from the same area who were matched for age, sex, and socioeconomic status.	20 Tobacco processors and 20 Control subjects	Not reported	Genotoxicity	Not reported
9	Baluka 2017	A Study on Reproductive Outcome, Health Problems and Haematological Profile in Rural Women Bidi Rollers of Reproductive Age in Jagitial	Case control study	Telangana	Rural area	Bidi rollers in the age range of 15 to 50 years from the villages of Ibrahimpatnam, Athmanagar, Ammakapet, Metpally, Godhur, and	230 Bidi workers and 200 Control subjects	Not reported	Skin problems; Neurological; Cardiovascular diseases; Musculoskeletal; Ophthalmological disorders; Gastrointestinal; Genito-urinary tract diseases; Respiratory;	Not reported

		District of Telangana State				Kathalapur in the Jagitial district were selected and control group consisted of equal numbers of bidi workers in the same age group belonging to the same socio-economic status and not occupationally exposed to chemical and physical agents			Endocrine; Anaemia and nutritional deficiency; Haematological disorders; gynaecological diseases	
10	Banu 2014	Pulmonary Functions among Bidi Rolling Workers of South India - A Cross Sectional Study	Cross-sectional study	Tamil Nadu	Southern and central part of Salem and widely distributed areas of Dharmapuri district	Men and women aged between 20-50 years rolling bidis at home and the workers in the bidi industry.	182 Bidi workers	Not reported	Respiratory diseases	Not reported

					employed at four private bidi industries	working for ⩾ 2yrs and exposed to tobacco dust > 8hours a day; 182 bidi workers				
11	Basu 2018	Morbidity Audit of Women Bidi Workers in an Urban Fringe of West Bengal, India	Cross-sectional study	West Bengal	Urban Fringe	Bidi workers residing in four wards, available during the survey time.	103 Bidi workers	The average (SE) monthly family income was Rs. 6177.67±270.90 .	Respiratory; Cardiovascular diseases; Gastrointestinal ; Ophthalmological disorders; Otolaryngology (ENT) diseases; Musculoskeletal ; Skin problems; Endocrine; Anaemia and nutritional deficiency;	Not reported
12	Bhat 2018	Assessment of oral mucosal conditions among bidi workers residing in bidi workers colonies in Karnataka, India	Cross-sectional study	Karnataka	Bidi workers' colonies	Subjects aged between 18 and 60 years involved in bidi making for 1 year or more and residing in	700 Bidi workers	Not reported	Oral problems	Nil

						bidi workers' colonies;				
13	Bhisey 1991	Biological monitoring of bidi rollers with respect to genotoxic hazards of occupational tobacco exposure	Case control study	Maharashtra	Not reported	Women bidi rollers free of tobacco habits and belonging to the same community, sharing common food habits were included they were designated as BR-K and BR-S 2 groups of bidi rollers which differed in living conditions. BR-K lived in the most populated area of Bombay, whereas people who were BR-S lived in a less	Bidi rollers (residing in densely populated area): 26. Bidi rollers residing in less congested area): 18; Control subjects (non-bidi rollers): 26	Not reported	Genotoxicity	Not reported

						crowded area with easy access to open spaces control group consisted of subjects (age- and sex-matched) non-bidi rollers without tobacco habits				
14	Bhisey 1992	Toxic effects of exposure to tobacco among bidi rollers	Case control study	Maharashtra	Not reported	Women bidi rollers who were non-users of tobacco. Non-users were chosen to clearly delineate the effects of occupational exposure. Control group consisted of	Bidi workers: 32 and Non-bidi workers/Control subjects: 26	Not reported	Genotoxicity	Not reported

						age-matched women who were not bidi rollers and had no tobacco habit.				
15	Bhisey 1999	Biological monitoring of bidi industry workers occupationally exposed to tobacco	Case control study	Mahara shtra	Non-mechanize d tobacco processing factory	Women workers from a non-mechanized tobacco processing factory located in Nipani, India, were monitored. Control group consisted of women workers from the same town, (matched for age, socio-economic status and habits) but who had never	Bidi workers: 46 and Non-bidi workers /Control subjects: 31	Not reported	Genotoxicity	This investigat ion was partly supporte d by a grant from the Indian Council of Medical Research.

						worked in this industry.				
16	Biswas 2021	Comparative analysis of respiratory health profile among female bidi and non-bidi workers in a district of West Bengal	Case control study	West Bengal	Households of wards of Kalyani municipality under Nadia district, West Bengal, India.	Adult women bidi workers who were permanent residents (more than six months) and working in the bidi industry for at least one year. Control group consisted of adult women who were not involved in bidi work.	Bidi workers: 30 Non-bidi workers: 30	Bidi workers contributed 1501-2000 INR in their family with mean [SD] contribution 1403.33 [768.11]	Respiratory disease	Nil
17	Buvaneswari 2008	Health problems of women bidi workers	Cross-sectional study	Tamil Nadu	Valluvar Nagar, Trichy	Bidi workers residing in Valluvar nagar	Bidi workers: 30	Majority of the respondents (63.3 percent) were earning from Rs.1000 to Rs.1500 per month from bidi rolling.	Musculoskeletal disease	Not reported

18	Chakraborty 2013	Bidi bundling as a means of women employment generation in backward rural area: a case study on char areas of bhagawangola-ii block, murshidabad district, west Bengal	Cross sectional study	West Bengal	Char areas from Bhagawangola-II block	Women, who were involved in bidi work and willing to answer the schedule;	55 Bidi workers	Not reported	Respiratory; Otolaryngology (ENT) diseases; Musculoskeletal; Mental health conditions; Ophthalmological disorders.	Not reported
19	Channegowda 2018	Effects of occupational tobacco exposure on mother and foetus in bidi rollers	Cohort Study	Karnataka	Tertiary care hospital	Pregnant women aged between 20 to 35 years with more than 28 weeks period of gestation and those who arrived for delivery at the same hospital.	500 Bidi workers	Not reported	Gynaecological diseases; Anaemia and nutritional deficiency; Disorders of new-born	Nil
20	Chattopadhyay 2006	A study to assess the respiratory impairments among the male	Case control study	West Bengal	Not reported	Male bidi workers were the study subjects,	Bidi workers: 51 Non-bidi workers/Control	Not reported	Respiratory disease	Nil

		bidi workers in unorganized sectors				control group consisted of those who were not directly engaged in bidi-making but were involved in some other jobs in the same area of study.	subjects: 56			
21	Chelimela 2018	Gynaecological Morbidity among women bidi workers in rural areas of Nizamabad district, Telangana	Cross-sectional study	Telangana	Rural villages	Women having more experience in bidi rolling and available at the time of the study;	560 Bidi workers	Not reported	Gynaecological disease	Not reported
22	Chithirairajan 2019	Ailments of women bidi labourers in Tirunelveli District, Tamil Nadu	Cross-sectional study	Tamil Nadu	Rural households	Bidi workers residing in households in rural areas.	Alangulam Village: 30 (women from well-off households: 10, women from poorer	Majority 37.3% of them earned less than Rs 3000, followed by 27.5% of the respondents earned between Rs 3000 to Rs 4500, and the remaining each	Neurological; Ophthalmological disorders; Musculoskeletal; Gastrointestinal; Otolaryngology (ENT) diseases; Skin problems;	Nil

							households : 20) Sankarnaga r village: 21 (women from well-off households : 9, women from poorer households : 12)	17.6% of the respondents earned between Rs 4500-6000 and more than Rs 6000. According to women well-off workers most of them earned Rs more than Rs 6000 which constitute 36.8% followed by 31.6% at Rs 4500-6000, 21.1% at Rs 3000-4500 and the least income level at Rs less than Rs 3000 which constitute 10.5%. Whereas women of poorer household, and majority more than half of them (53.1%) earned the monthly income level as Rs less	respiratory diseases	

								than 3000, followed by 31.2% at Rs 3000-4500, 9.4% at the Rs 4500-6000 and rest of them 6.3% earned at Rs more than 6000.		
23	Chodhury 2012	Current trends in girl child labour: A case study of homebased bidi workers of Malda and Murshidabad districts of West Bengal India	Cross-sectional study	West Bengal	Two sub-divisions from the Murshidabad district viz. Jangipur and Berhampore and the southern part of the Malda District (the district of Malda consists of only two sub-divisions – Englishbazar and Chanchal	Bidi rolling children (girl child) up to the age of 18 years.	Families of bidi workers: 360 and children bidi rollers: 278	A roller can make about 1,000 bidis a day and is paid ` 65 to ` 80 for the day’s work. The children work from 7 a.m. to 10 p.m. every day on a daily wage of ` 50 to ` 55.	Respiratory diseases; Musculoskeletal diseases	Not reported

					sub-division); blocks of Murshidabad district were Raghunathganj-I, Suti-II of Jangipur sub-division and Hariharpara, of Berhampore sub-division. Whereas in case of Malda district the blocks are Kaliachak-I, Manikchak, and Old Malda					
24	Das 2013	A study on occupational health hazards among women bidi workers of	Cross-sectional study	West Bengal	Two villages and two municipal wards in Jangipur	Women bidi-workers belonging to the Muslim community were	92 Bidi workers	Worker's Contribution (%) to Family's Total Monthly Income- ≤20%= 40	Musculoskeletal; Respiratory; Gastrointestinal; Ophthalmological disorders;	Not reported

		Murshidabad West Bengal			Sub-division of Murshidabad district	married and without tobacco smoking and chewing habits residing in different areas of Jangipur sub-division in Murshidabad district		20-30%= 34<br>>30%= 18	Gynaecological diseases; Neurological diseases	
25	Dhotre 2017	A comparative study of lipid profile and haematological parameters in women bidi rollers and non-bidi rollers in rural population of Solapur, Maharashtra	Case control study	Maharashtra	Rural Medical College, Hospital and Research Centre	Women bidi rollers aged between 28-60 years had been in this profession for at least 5 years and not having the habit of tobacco smoking/ chewing. Control group consisted of non-bidi roller women aged between 28-	Women bidi Rollers: 50; Non-bidi roller women/control subjects: 50	Not reported	Haematological disorders; Lipid profile	Not reported

						60 years not suffering from any chronic diseases, or taking any medication and not having the habit of tobacco chewing or smoking.				
26	Fredi Moses 2011	Evaluation of occupational exposure to toxic metals using fingernails as biological indicators	Cross-sectional study	Tamil Nadu	Match, cracker and bidi factories	Women workers aged between 15-50 years from the match, cracker and bidi factories residing in and around Virudhunagar district.	20 Fingernails of bidi workers	Not reported	Genotoxicity	Tamil Nadu State Council for Science and Technology, Chennai
27	Gautam 2015	Genotoxic effects of tobacco dust exposure on bidi rollers	Case control study	Madhya Pradesh	Not reported	Women bidi rollers of different ages and exposures.	Bidi workers: 34;	Not reported	Genotoxicity	UGC (India) for funding the research

						Control group consisted of subjects same as the study group and who were neither smokers nor tobacco chewers.	Control subjects: 30			through a major research project [F. No. 39643/2010(SR) dated 10-01-2011].
28	Ghatak 2017	Ground Realities of Bidi Workers In West Bengal	Qualitative study	West Bengal	Bidi industry; hospital	Women bidi workers and adolescent girls residing in Murshidabad and Malda districts; officials of bidi companies, labour department, health department, trade union office bearers, and three NGOs: ASHA (Association	6 Focus group discussions (bidi workers and adolescent girls) and 9 stakeholder interviews.	Wage being paid was Rs 126 per 1000 bidis. Factories explain this as Rs 105 + Rs 21 for bonus. Workers are paid for 950 bidis and not a 1000	Respiratory diseases; Musculoskeletal diseases. Genito-urinary tract infections. Gynaecological diseases; Ophthalmological diseases	Not reported

						for Social and Health Advancement), Marfat and Reach India Trust - working for the rights of bidi workers in the state.				
29	Ghosh 1985	Occupational Health Problems among Tobacco Processing Workers: A Preliminary Study	Case control	Gujarat	Factories	Subjects working in bidi factories and occupationally exposed to any form of tobacco during their day work; Control group consisted of subjects who were not occupationally exposed to any form of tobacco during their work and belonged to the same	100 Bidi workers; Control subjects: 50	Not reported	Gastro-intestinal problems; Musculoskeletal; Neurological	Not reported

						socioeconomic group.				
30	Gladstone 2008	Infant morbidity in an Indian slum birth cohort	Cohort study	Tamil Nadu	Urban slums	Children of pregnant women residing in a brick-built house with five or more rooms intending to remain in the area for 3 years;	New-borns: 452; Pregnant women: 914	Not reported	Respiratory diseases; Morbidity	This work was supported by the Welcome Trust Trilateral Initiative for Infectious Diseases, grant no. 063144.
31	Gopal 2000	Health of Women Workers in the· Bidi Industry	Case study	Tamil Nadu	Not reported	Bidi workers residing in block of the Tenkasi Taluk.	Bidi workers: 237	Not reported	Musculoskeletal; Respiratory; Gastro-intestinal	Not reported
32	Govekar 1992	Elevated urinary thioether excretion among bidi rollers exposed occupationally to processed tobacco	Case control study	Maharashtra	Hospital	Women bidi rollers and non-bidi rollers with no tobacco habit and those habituated to the use of masheri and/or chewing of	Bidi workers: 69, non-bidi workers/Controls: 83, Healthy volunteers: 98	Not reported	Genotoxicity	Not reported

						betel quid with tobacco were selected and subdivided into 2 groups, a group of healthy young volunteer's aged between 17-18 years of both sexes who had no tobacco or alcohol habits.				
33	Hegde 2015	A study to evaluate respiratory symptoms and lung function impairment in Bidi workers and usefulness of protective mask in reducing respiratory symptoms.	others	Karnataka	Not reported	Healthy male and female subjects aged between 20 to 40 years who had been exposed to tobacco dust for minimum three years.	Subjects exposed to tobacco dust: 32; Subjects not exposed to tobacco dust: 32	Not reported	Respiratory diseases	Not reported

34	Islam 2021	Occupational Health Hazard of Female Bidi Workers in Rural West Bengal: A Case Study	Cross-sectional study	West Bengal	Rural Households	Women bidi workers residing in the Murshidabad district;	120 Bidi workers	Approximately 110 rupees per day	Musculoskeletal; Respiratory; Gastrointestinal; Ophthalmological disorders	Not reported
35	Jael 2016	Effects of Occupational Exposure to Tobacco Dust in Women Bidi Rollers	Case control study	Telangana	Warangal, Nizamabad & Adilabad districts	Women bidi workers having experience ranging from 16-66 years and who worked for 8 h/per day;	Women bidi rollers: 182; Age matched subjects/controls: 182	Not reported	Respiratory disease; Ophthalmological disorders; musculoskeletal; Cardiovascular diseases; Neurological disease	Ministry of Environment and Forests [MOEF], Govt. of India, New Delhi
36	Joseph 2016	Association between occupational history of exposure to tobacco dust and risk of carcinoma cervix: A case-control study	Case control study	Karnataka	Two tertiary care hospitals	Incident cases of carcinoma of the cervix that had been histologically verified at the corresponding centres. A 5-year age group matched	Exposed subjects (tobacco use): 239, Non-exposed subjects/Control subjects: 239	Not reported	Oncological conditions	Manipal University research grants

						control from the same hospital admitted with a condition not associated to tobacco use was selected.<br>By ruling out the symptoms and signs controls were ascertained for absence of carcinoma cervix				
37	Joshi 2013	An epidemiological study of occupational health hazards among bidi workers of Amarchinta, Andhra Pradesh	Cross-sectional study	Andhra Pradesh	Rural area	Subjects involved in bidi rolling;	470 Bidi workers	Not reported	Musculoskeletal diseases; respiratory diseases; Neurological problems; Gastro-intestinal problems; Skin disorder	Not reported

38	Kamath 2017	Prevalence of bronchial asthma among school-going children in Mangalore, South India	Cross-sectional study	Karnataka	Two private schools	Children in the age group of 6 to 15 years, studying in classes I-X, and present in class on the day of distribution.	1011 Children of the bidi workers	Not reported	Respiratory diseases	Nil
39	Kanagavalli 2015	Common Health Hazards among Bidi Workers	Cross-sectional study	Karnataka	Rural community	Women involved in bidi rolling aged between 20-60 years residing in Kompadavu area of Mangalore and willing to participate in the study	30 Bidi workers	Maximum numbers of women earning below Rs 3000 /month as their family income	Musculoskeletal diseases; Gynaecological diseases; Neurological diseases; Anaemia and Nutritional disorder; Gastro-intestinal diseases;	Not reported
40	Kanipakam 2021	Association between Occupational Exposure to Tobacco Dust and Absolute Telomere Length: A Cross-	Case control study	Tamil Nadu	Tiruchirappalli bidi sector	Women bidi workers aged between 20 to 35 years, whose duration of employment	Bidi workers: 20; Non-bidi workers: 20	Not reported	Genotoxicity	Nil

		sectional Study on Female Bidi Workers				should be >1 year and <3 years with no other comorbidities or diseases that could affect alterations. Control group consisted of women non-bidi workers aged between 20–35 years, and whose family members were also not involved in bidi rolling industry.				
41	Kaup 2017	Occupational exposure to unburnt tobacco and potential risk of toxic optic neuropathy: A cross-sectional study among	Cross-sectional study	Karnataka	Rural area	Subjects involved in bidi rolling for at least one year and willing to participate in the study.	365 Bidi workers	Not reported	Ophthalmological disorders	This study was conducted as a part of Indian Council of Medical Research-

		bidi rollers in selected rural areas of coastal Karnataka, India								Short Term Studentship (ICMR-STS). One of the authors (AN) has been granted a scholarship of INR 10,000. However, authors received no specific funding for this work.
42	Kawale 2020	Cross sectional study of health problems in female bidi rollers in Telangana, India.	Cross-sectional study	Telangana	Bidi factories	Women who were present during the visit to the bidi factory and willing to participate in the interview process.	400 Bidi workers	Not reported	Musculoskeletal diseases; respiratory diseases; Gastro-intestinal problems; Skin problem; Endocrine disorders	Nil

43	Khanna 2014	Tobacco dust induced genotoxicity as an occupational hazard in workers of bidi making cottage industry of central India	Case control study	Madhya Pradesh	Not reported	Healthy women bidi workers aged between 25 to 68 years and their occupational exposure ranged from 13 to 58 years. Control group consisted healthy volunteers aged between 25 to 65 years. selected bidi workers and controls were neither smokers nor did they indulge in tobacco chewing.	Healthy female bidi workers: 31, Non-bidi workers/Control subjects: 30	Not reported	Genotoxicity	UGC (India) for funding the research through a major research project [F. No. 39-643/2010 (SR) dated 10-01-2011].
44	Kollur 2015	Status of bidi rollers in urban slum area of	Cross-sectional study	Karnataka	Urban slum area adopted	All residents of the Community	29 Bidi workers	They get Rs. 100/- for rolling 1000 bidis	Endocrine; Cardiovascular	No funding sources

		Dharwad city, India			under Urban Health Training Centre (UHTC)	adopted under UHTC who have been engaged in bidi rolling for at least the past six months.			diseases; Musculoskeletal	
45	Krishna 2018	Morbidity profile of Bidi workers in the Urban Field Practice Area of a Medical College in Tumakuru	Cross-sectional study	Karnataka	Urban slum in the field practice area	All bidi workers of both sexes who have been employed for at least six months and have been living in an urban slum in a medical college's field practise area.	120 Bidi workers	Income from bidi rolling was Rs. 1000 [IQR:6000-12000]	Endocrine disease; cardiovascular diseases; Respiratory; Musculoskeletal; Gastrointestinal; Neurological; Ophthalmological disorders; Skin disease; Mental health conditions	Nil
46	Kumar 2019	Study of Morbidity Pattern among Women Bidi Rollers Residing	Cross-sectional study	Karnataka	Urban field practice area	women who have been Bidi rollers for at least six months	120 Bidi workers	Not reported	Musculoskeletal; Ophthalmological disorders; Respiratory; Skin problems;	Nil

		in Urban Area of Mangalore							cardiovascular diseases; Endocrine; Gastrointestinal problems; Neurological; Anaemia and nutritional deficiency	
47	Kuruvila 2002	Occupational dermatoses in Bidi rollers	Case control study	Karnataka	Bantwal camp (study group); Skin OPD, District Wenlock Hospital (Control group)	Bidi workers visiting the Bantwal camp (study group); Individuals matched by age and sex visiting the District Wenlock Hospital's Skin OPD (control group)	91 Bidi workers; Control subjects: 33	Not reported	Skin problems	Not reported
48	Latha 2018	A study on the socio-economic status of women bidi workers in Tirunelveli district, Tamil Nadu	Cross-sectional study	Tamil Nadu	Not reported	Women bidi workers	100 Bidi workers	Family income (Rs) : Less than 3000 (8%); 3001-5000 (16%); 5001-7000 (44%);	Musculoskeletal; Respiratory; Ophthalmological disorders	Not reported

								Above 7000 (32%)		
49	Logu 2021	A Study on Problems and Prospects of Women Bidi Workers in Mudukulathur, Ramanathapuram District	Cross-sectional study	Tamil Nadu	Households of Mudukulathur	Bidi workers residing in the households of Mudukulathur	250 Bidi workers	The analysis and test reveals that there is close relationship between income and expenditure pattern. Most of the women bidi worker expenses a small percentage of their income, if the income will increase, expenditure will also be increased. So there is a close relationship between income and expenditure of women bidi rolling worker. 20.8 per cent of the respondents are problem faced by low wages. 9.2 per	Musculoskeletal; Neurological; Respiratory; Gastrointestinal illness; Gynaecological problems; Others (swelling and fungal disease)	Not reported

								cent of the respondents are problem faced by not enough income		
50	Madhusudan 2014	Occupational health profile of bidi workers in coastal Karnataka	Cross-sectional study	Karnataka	Urban field practice area	Bidi workers (of both sexes) who have been employed for at least six months and residing in Urban field practice area.	439 Bidi workers	Average monthly bidi income was Rs.882.6 households monthly per capita income was Rs.1000 – 1999.	Musculoskeletal; Ophthalmological disorders; cardiovascular diseases; Respiratory; Endocrine diseases; Skin diseases; Oral problems; Others	Nil
51	Mahimkar 1995	Occupational exposure to bidi tobacco increases chromosomal aberrations in tobacco processors	Case control study	Maharashtra	Households	Subjects working in tobacco processing factory	67 Bidi workers	Not reported	Genotoxicity	Not reported
52	Mandelia 2014	Effects of Occupational Tobacco Exposure on Foetal Growth, among Bidi Rollers in	Case control study	Karnataka	Hospital	Women between the ages of 20 and 35 admitted to the post-natal ward, who had full-	102 Bidi rollers; non-bidi rollers/Control subjects: 102	Not reported	Disorders of new-born	The study was supported by a research grant from Indian

		Coastal Karnataka				term, normal, vaginal deliveries in the hospital and who work as bidi rollers made up the study group, while the control group consisted of those who don't work as bidi rollers.				Council of Medical Research (ICMR), under STS-2010, awarded to Dr. Chetan Mandelia. (Grant # ICMR-STS-2010-00349)
53	Mascarenhas 2019	Impact on bidi rolling on lung functioning- A Cross Sectional study.	Cross-sectional study	Karnataka	Households	Bidi workers who had been rolling bidi and exposed to tobacco dust for more than two years;	66 Bidi workers	Not reported	Respiratory disease	Nil
54	Mishra 2014	Nimble Fingers on Bidis: Problems of Girl Child Labour in Sambalpur and Jharsuguda	Qualitative study	Orrisa	Households	Bidi workers residing in the households of Sambalpur and	3 Women bidi workers	Bidi workers of Rengali claimed that they get `Rs. 40 per 1,000 bidis.	Respiratory diseases; Musculoskeletal diseases; Ophthalmological disorders	Not reported

						Jharsuguda district				
55	Mittal 2018	Ocular manifestations in bidi industry workers: Possible consequences of occupational exposure to tobacco dust	Cross-sectional study	Tamil Nadu	Tertiary eye care centre in a rural area	women who were actively engaged in bidi-rolling and presenting in the outpatient department with ocular complaints;	310 Bidi workers	Not reported	Ophthalmological disorders; Neurological diseases; Skin diseases; Gynaecological diseases; Respiratory; diseases Musculoskeletal; Endocrine; Cardiovascular diseases	Not reported
56	Mohandas 1980	Bidi Workers in Kerala: Conditions of Life and Work	Cross-sectional study	Kerala	Not reported	Bidi workers residing in Malabar region and Trichur district of Kerala.	100 Bidi workers	91 per cent of the workers had incomes of less than Rs 200 per month. The income of 70 per cent of them ranged. between Rs 100 and Rs 200, while 20 per cent has less than Rs 100 per month. Home based: 70 per cent earned less than Rs 100 per month.	Respiratory diseases; Otolaryngology (ENT) diseases; Neurological diseases; Gastrointestinal diseases; Musculoskeletal diseases; Skin problems	Not reported

								40 per cent for the factory-based earned less than Rs 100 per month		
57	Mukherjee 2014	A Study on health profile of bidi workers in West Bengal, India	Cross-sectional study	West Bengal	Gram Panchayats	From the district, one Block was chosen at random. Six Gram Panchayats total were selected for this Block. To ensure that every social class is fairly represented, bidi workers were chosen at random from each Gram Panchayat.	120 Bidi workers	Not reported	Respiratory; Oral problems; Skin problems; Gynaecological diseases; Musculoskeletal diseases; Disorders of new-born; Otolaryngology (ENT) diseases; Gastrointestinal; Genito-urinary tract diseases; cardiovascular diseases; Neurological diseases; Oncological conditions	Not reported
58	Mukhopadhyay 2008	Caught in a death trap the story of bidi rollers of West Bengal and Gujarat	Mixed method study	West Bengal; Gujarat	Households	Bidi workers residing in Murshidabad and Anand district	1000 Bidi workers (500 Murshidabad district. 500 Anand district)	Murshidabad district 69% less than Rupees 1,000 per month, 28% reported their income to be within the range	No disaggregated data reported - Qualitative part reports diseases	Not reported

								of Rupees 1,000 to 2,000 per month and 3.2% earned more than Rupees 2000 per month; Although normal rate is Rupees 40 there are reports of some rollers making only Rupees 25 to 35 per 1000 bidis; The schedule of payment is mostly on a weekly basis. Some get paid daily and some receive payment on a monthly basis.<br><br>Anand District: Earning: 82.2% earned less than Rs 1,000 per month. 17.40% reported their income per month to be		

								between Rs 1,000 to 2,000 while 0.40% reported they earned more than Rs 2,000 per month. The going rate for bidi rolling here is Rs. 60 for 1000 bidis. However, they are demanding that it be raised to Rs 65		
59	Murugan antham 2019	Tobacco Exposure and its Possible Electrocardiographic Changes in Female Bidi Rollers in Tirunelveli District	Cross-sectional study	Tamil Nadu	Not reported	Women bidi rollers, 20 to 50 years old, who have been involved in bidi rolling for at least 5 years. It was confirmed that they had never smoked and were free of any other pre-existing conditions.	35 Bidi workers	Not reported	Cardiovascular diseases	Nil

60	Nag 1986	Occupational stresses on women engaged in making bidi	Cross-sectional study	Gujarat	Households	Women working in the bidi-making household industries.	178 Bidi workers	Not reported	Musculoskeletal diseases; Neurological diseases; Ophthalmological disorders; cardiovascular diseases; Gastrointestinal problems; Respiratory diseases	Not reported
61	Nakkeeran 2010	A Study on Occupational Health Hazards among Women Bidi Rollers in Tamil Nādu, India	Cross-sectional study	Tamil Nadu	Households	Women bidi rollers who don't smoke or chew tobacco.	388 Bidi workers	Not reported	Skin problems; Ophthalmological disorders; Musculoskeletal diseases; Gastrointestinal diseases; Neurological diseases; respiratory diseases; Anaemia and nutritional deficiency;	Not reported
62	Palande 2018	A study of health problems of women bidi rollers in Ahmednagar district with special	Cross-sectional study	Maharashtra	Households	Women bidi workers.	303 Bidi workers	Not reported	Neurological diseases; Musculoskeletal diseases. Respiratory diseases;	Not reported

		reference to Akole and Sangamner tahsils of Ahmednagar district Maharashtra.							Gastrointestinal problems	
63	Pandian 2021	Occupational Health Hazards among Women Bidi Workers in Srivaikuntam Taluk of Thoothukudi District	Cross-sectional study	Tamil Nadu	Households	Women involved in Bidi-rolling.	120 Bidi workers	Not reported	Respiratory diseases; Otolaryngology (ENT) diseases; Neurological; Musculoskeletal diseases; Fever; Ophthalmologic al disorders	Not reported
64	Patel 2017	An anthropological study of morbidity pattern of female bidi workers residing in district Bilaspur, Chhattisgarh (India)	Cross-sectional study	Chhattis garh	Urban and semi-urban Households	Subjects actively involved in Bidi rolling.	424 Bidi workers	Income: minimum 60 Rs and maximum 89 Rs. per day.	Cardiovascular diseases; Musculoskeletal diseases; Neurological diseases; respiratory diseases; Gastrointestinal diseases; Ophthalmologic al disorders; Mental health conditions	Not reported
65	Prakash 2012	Prevalence and Risk Factors for Respiratory	Cross-sectional study	Rajastha n	Not reported	Bidi workers belonging to low-income	Bidi workers using	Not reported	Respiratory diseases; Anaemia and	Not reported

		Manifestations in Female Bidi Workers of Ajmer				group and having long working hours. They were further divided into 2 major groups- tobacco users and non- tobacco users.	tobacco: 100, Bidi workers not using tobacco :100		nutritional deficiency; Gastro-intestinal; Others (swelling)	
66	Prakash 2013	Association between occupational tobacco exposure of health hazards in women labourers of bidi-industry of Ajmer	Cross-sectional study	Rajasthan	Not reported	Women bidi workers actively involved in Bidi-rolling.	200 Bidi workers	Not reported	Neurological diseases; Musculoskeletal; Skin problems; Ophthalmological disorders; Gastrointestinal problems; Respiratory; Otolaryngology (ENT) diseases	Not reported
67	Priya 2018	Health hazards among bidi rollers in Dakshina Kannada District of Karnataka: A case study of women workers	Mixed method study	Karnataka	Household of Dakshina Kannada district	Bidi workers residing in households of Dakshina Kannada.	Female bidi workers: 120	Not reported	Ophthalmological disorders; Gastrointestinal problems; Neurological disorders; respiratory diseases; Skin Problems;	Not reported

									Musculoskeletal diseases; Fever	
68	Raju 2015	Comparative Study on the Health Problems between Bidi Rolling Women and non-Bidi Rolling Women of a Selected Community at Mangalore	Case control study	Karnataka	Urban community areas of Mangalore (Basavanagar and Devinagar)	Women bidi workers and non-bidi workers aged between 18-45 years.	Bidi workers: 50 Non bidi workers/controls: 50	Not reported	Musculoskeletal diseases; respiratory diseases; neurological problems; skin problems;	No funding was required
69	Ranjitsingh 1995	Occupational Illness of Bidi Rollers in South India	Cross-sectional study	Tamil Nadu	Households	Bidi workers residing in different parts of Tirunelveli district.	450 Bidi workers	Income: Rs 30 per day; 61% below 1000rs (monthly income);	Respiratory diseases; Otolaryngology (ENT) diseases; Skin problems; Gastrointestinal problems; Gynaecological; Musculoskeletal; Oncological conditions; Anaemia and nutritional deficiency	Not reported
70	Rao 2020	Maternal Occupational Tobacco Exposure and New-born Umbilical Cord	Case control study	Karnataka	Not reported	Neonates born to bidi workers, Small for gestational age (SGA)	64 Neonates born to bidi Workers; 64 small for gestational	Not reported	Disorder of New-born	Nitte deemed to be university Faculty

		Serum Leptin Concentration				neonates and term appropriate for gestational age (AGA) new-borns with no maternal occupational tobacco exposure or history of smoking were enrolled.	age new-borns (SGA) and 57 Term appropriat e for gestational age (AGA) new-borns.			research grant
71	Rao 2020a	Occupational Morbidity of Women Bidi Workers in Telangana	Cross-sectional study	Telanga na	Rural Households	Women bidi workers living in rural areas of Nizamabad district, Telangana.	560 Women bidi workers	Proportion of income from bidi making: <1/4th: 121 (22.92); 1/4th-½: 298 (56.44); ½-3/4th: 26 (4.92); 3/4th-full: 83 (15.72) Average total income of the family and per-capita income were Rs. 4953 and Rs. 1240	Musculoskeletal diseases; Gynaecological; Respiratory diseases; Ophthalmologic al disorders; Neurological disorders	Nil

72	Rayhan 2021	Nutritional status and concomitant factors of stunting among pre-school children in Malda, India: A micro-level study using a multilevel approach	Cross-sectional study	West Bengal	Rural and urban areas from the 12 selected PSUs (primary sampling units), which were nested within the three selected blocks and a municipality in the Malda district. The villages in rural areas and wards in urban areas were considered as the PSUs	Women with at least one pre-school-aged child (aged between 36 to 59 months old) living in the households. If there was more than one child in the age group of 36 to 59 months in a selected household, than only one child was selected following the alphabetic order of child's name.	Children/mothers: 731	Not reported	Nutritional deficiencies	The authors received no specific funding for this work.
73	Rout 2017	Poverty and Health Status of Bidi Workers in Andhra Pradesh	Mixed method study	Telangana	Households engaged in bidi rolling in Karimnagar district -	Households having at least one bidi worker involved in the bidi	1403 Bidi workers	The share of income from bidi rolling was `23,549.97. A worker earned `1,760 per	Gastrointestinal diseases; Respiratory diseases; Skin Problems;	Not reported

					two towns, six mandal headquarters (semi-urban) and six villages of Karimnagar.	rolling industry for six months or more during the previous year.		month from bidi rolling. As bidi rolling largely involved women rollers, their share constituted 77.3% of the aggregate income from bidi rolling.	Musculoskeletal diseases	
74	Russell 2022	Implementing FCTC Article 17 Through Participatory Research With Bidi Workers in Tamil Nadu, India	Qualitative study	Tamil Nadu	Households	Women involved in bidi rolling and residing in hamlets and informal settlements in Tirupattur and Vellore.	46 Bidi workers	Daily Income: Rs 170 [c. US$ 2.25] per day	neurological problems; gastro-intestinal problems; Cardiovascular	Global Challenges Research Fund networking grant (GCRFNG R3\1224) administered by the UK Academy of Medical Sciences
75	Sabale 2012	Working condition and health hazards in bidi rollers residing in the	Cross-sectional study	Maharashtra	Urban slums of Mumbai	Bidi workers currently involved in the bidi rolling.	52 Bidi workers	The average daily wage is Rs. 40.	Musculoskeletal diseases; Ophthalmological disorders; Otolaryngology (ENT) diseases;	Nil

		urban slums of Mumbai							respiratory diseases; neurological problems; gastro-intestinal problems; skin problems; gynaecological problems; Others-weakness, fatigue	
76	Sardesai 2007	Tabacco handling by pregnant bidi workers: As hazardous as smoking during pregnancy	Case control study	Maharashtra	Women reporting to labour wards for delivery in hospital	Women bidi workers reporting to labour wards for delivery in hospital.	Phase 1: Bidi workers: 276 Phase 2: Bidi workers at term 100. Phase 3: Bidi workers pregnant or otherwise: 76	Not reported	Gynaecological diseases; disorders of new-born	Not reported
77	Sarkar 2004	Women workers in bidi rolling	Cross-sectional study	West Bengal	Households of Cooch Behar-Sadar, Dinhata and Tufangang	Bidi workers residing in the households of Dinhata and Tufangang	200 Bidi workers	The wage rate for the factory workers in Cooch Behar district varies from Rs.30 to Rs.33.50 per	Musculoskeletal diseases; Ophthalmological disorders; respiratory diseases;	Not reported

						towns in Cooch Behar district, West Bengal.		1,000 bidis, while contract workers are paid of Rs. 24 to Rs.28 per 1000 bidis. Most of the workers have incurred on an average expense of Rs. 1.50 to Rs. 2 per 1,000 bidis out of their own pocket due to deficiency of raw materials and rejection of defective bidis which, in tum, results in decrease in their wage rate.		
78	Sen 2007	Effect of working condition on health of bidi workers: A study of Sagar district of Madhya Pradesh.	Cross-sectional study	Madhya Pradesh	Households	Bidi workers residing in Khurai block.	70 Bidi workers	Rs 23-32 for 1000 bidis	Musculoskeletal diseases; Ophthalmologic al disorders; Otolaryngology (ENT) diseases; Gastrointestinal ; Neurological; Respiratory	Not reported

79	Sharma 2018	Recasting Language of Work: Bidi Industry in Post-colonial Central India	Qualitative study	Madhya Pradesh	Households	Women workers involved in home-based bidi making	Group interviews; 3 bidi workers	Not reported	Mental health conditions	Not reported
80	Shatrugna 2008	Relationship between women's occupational work and bone health: a study from India	Cross-sectional study	Telangana	Urban slum, Addagutta (Hyderabad, India)	Adult women residing in large urban slum, Addagutta (Hyderabad, India), from three occupations (sweepers, construction workers and bidi rollers) aged between 30 to 60 years and involved in their occupation for minimum of 5 years.	Bidi workers: 48 Non- bidi workers/ involved in other occupation: 158	Not reported	Musculoskeletal disease	Indian Council of Medical Research.
81	Shenoy 2020	Pregnancy Outcome in Occupational Tobacco	Cohort study	Karnataka	Hospital-based	Women aged between 19 to 35 years with	Exposed cohort- 177 Unexposed cohort- 354	Not reported	Gynaecological diseases	Indian Council of Medical Research

		Exposure: A Cohort Study from South India				singleton pregnancies with no antecedent chronic illnesses, and were exposed to bidi rolling for at least 1 year before and continued into pregnancy.				
82	Shukla 2011	Working condition: A key factor in increasing occupational hazard among bidi rollers: A population health research with respect to DNA damage	Cross-sectional study	Madhya Pradesh	Not reported	Bidi workers aged between 18-65 years working under confined exposure or ventilated surroundings.	72 Bidi workers	Not reported	Genotoxicity	Nil
83	Singh 2014	Occupational Health Problems Amongst Women Bidi	Cross-sectional study	Uttar Pradesh	Households of Bundelkhand region	Women bidi workers with very high percentage of labour	216 Women bidi workers	The average monthly income of women bidi rollers was Rs. 300-1500/-.	Ophthalmological disorders; respiratory diseases; neurological	Not reported

		Rollers in Jhansi, Bundelkhand region, Uttar Pradesh				force in the bidi rolling at their homes and with no smoking or tobacco chewing habits			diseases; skin problems. Genito-urinary tract diseases; musculoskeletal diseases.	
84	Singh 2018	Socio-economic, Health and Working Conditions of Child Bidi Rollers in Bidi Industry	Cross-sectional study	Maharashtra	Bidi manufacturing factories	Children (both male and female) in the age group of 9 to 14 years involved in bidi rolling from various bidi manufacturing factories in Solapur city	118 Bidi workers	(55%) are earning Rs. 1000 to Rs. 3000 per month, 20% earning above Rs. 3000 per month. Around 25% child bidi rollers get a meagre monthly income of less than Rs. 1000.	Neurological; Musculoskeletal diseases; Otolaryngology (ENT) diseases; respiratory diseases; Gastrointestinal diseases	Not reported
85	Singh 2021	Assessment of Periodontal Status and Treatment Needs among Bidi Factory Workers	Cross-sectional study	Bihar	Bidi factory	Bidi workers aged between 19 to 60 years	1000 Bidi workers	Not reported	Oral problems	Nil
86	Srinivasan 2013	Occupational Health Problems Faced	Cross-sectional study	Tamil Nadu	Not reported	Women who were head of the family,	50 Bidi workers	Not reported	Oncological conditions;	Not reported

		By Female Bidi Workers at Khajamalai, Trichy District, Tamil Nadu				involved in Bidi work and willing to answer the schedule			endocrine diseases. Musculoskeletal diseases; cardiovascular diseases; respiratory diseases	
87	Sudina 2015	Occupational health problems among bidi workers of Udupi District in South India	Cross-sectional study	Karnataka	Athrady, Marne, and Hirebettu villages	Subjects involved in bidi rolling aged between 20 to 70 years with an experience of more than one year and residing in the villages of Udupi District;	200 Bidi workers	92.5% of the subjects were having a monthly income less than Rs.5,000.	Musculoskeletal diseases; respiratory diseases; Skin and Ophthalmological disorders	Not reported
88	Sundaramoorthy 2013	Clinical, Cytogenetic and CYP1A1 exon-1 Gene Mutation Analysis of Bidi Workers in Vellore Region, Tamil Nadu	Case control study	Tamil Nadu	Not reported	Bidi workers in the age group of 28-67 years and had work exposure for 8-60 years. Control group consisted of age and sex	Bidi workers: 27 Non-bidi workers/ Control subjects: 27	Not reported	Genotoxicity; Musculoskeletal diseases; Ophthalmological disorders; respiratory diseases; neurological diseases; skin problems;	VIT University

						matched subjects.			cardiovascular diseases.	
89	Surya Prabha 2018	Morbidity Profile among Women Bidi Workers in the Urban Slum of Kurnool Town, Andhra Pradesh	Cross-sectional study	Andhra Pradesh	Household of Budhawarpet, urban slum of the Kurnool town	Women Bidi workers working from at least 6 months.	203 Bidi workers	Not reported	Musculoskeletal diseases; Ophthalmological disorders; respiratory diseases; neurological diseases; skin problems; gynaecological problems	Not reported
90	Swami 2006	Absorption of Nicotine Induces Oxidative Stress Among Bidi Workers	Case control study	Maharashtra	Not reported	Women bidi workers between the age group of 20-60 years clinically diagnosed for any disease leading to oxidative stress and only those having no concurrent or past history of diseases. Control group	Female bidi workers: 90, Non-bidi workers/control subjects: 30	Not reported	Genotoxicity	Not reported

						consisted of healthy women from same socio-economic status, with no history of smoking and exposure to any occupational dusts all the subjects were further sub-divided into 3 groups based on their experience of bidi rolling.				
91	Tabassum 2018	Occupational tobacco exposure and health risks of Women Bidi rollers in Bihar Sharif (Nalanda)	Cross-sectional study	Bihar	Not reported	Women workers of the families, who were directly involved in bidi work and wiling to answer the schedule	200 Bidi workers	Not reported	Neurological diseases; Musculoskeletal diseases; Skin problems; Ophthalmological disorders; Gastrointestinal; Respiratory diseases;	Not reported

						were involved.			Otolaryngology (ENT) diseases	
92	Thabassum 2013	A study on women bidi at Vellore district in Tamil Nadu	Cross-sectional study	Tamil Nadu	Areas of Ranipet Vellore town, Gudiyatham and Katpadi	Women involved in bidi rolling and residing in areas of ranipet Vellore town, Gudiyatham and Katpadi	100 Bidi workers	Wage per week in Rs.<br>Rs 50 - 12%;<br>Rs 51-150 - 30%<br>Rs 151-300-39%<br>Rs 301-540-13%<br>Above 451- 6%	Anaemia and nutritional deficiency; Ophthalmological disorders; Gastro-intestinal problems; Neurological diseases; respiratory diseases; Skin Problems; Musculoskeletal diseases.	Not reported
93	Thomas 2015	A cross sectional study on health profile of female bidi rollers in a rural area in Mangalore	Cross-sectional study	Karnataka	Rural field practice area	Women who have been working as bidi rollers for at least six months	100 Bidi workers	Not reported	Respiratory diseases; Otolaryngology (ENT) diseases; Musculoskeletal diseases; Gastrointestinal diseases; Neurological; Mental health conditions; Ophthalmological disorders; Skin problems;	Not reported

94	Yasmin 2010	Occupational Health Hazards in Women Bidi Rollers in Bihar, India	Case control study	Bihar	Urban households	Women bidi rollers without tobacco smoking/chewing habit not suffering with any serious disease and were not on any medications. Control group consisted of women (mainly vegetable/fish sellers), with no history of occupational exposure to tobacco, and without any tobacco smoking/chewing habits, but belonging to the same age group and	Bidi workers: 197 Non-bidi workers/ control subjects: 61	The average monthly income of women bidi rollers was Rs. 378 (8.2 $), while that of controls was Rs. 650 (14.2 $).	Respiratory diseases; Musculoskeletal diseases; Ophthalmological disorders ; Gastro-Intestinal problems; Neurological; Gynaecological diseases; Haematological disorders	University Grants Commission for providing financial support under the scheme of Basic Scientific Research

						socio-economic status as the bidi workers.				
95	Yunus 2019	” Tobacco” – The silent slayer for oral premalignant lesions/ conditions among bidi rolling workers of Durg City, Chhattisgarh, India: A cross-sectional study	Cross-sectional study	Chhattis garh	Registered and licensed factories	Subjects working in bidi making factories and were present at the time of study.	185 Bidi workers	Not reported	Oral problems	Nil
