# Supplementary material for: Occupational health hazards of bidi workers and their families in India: a scoping review
Source: BMJ Glob Health. 2023 Nov 2;8(11):e012413. doi: 10.1136/bmjgh-2023-012413 (PMC10626877; doi:10.1136/bmjgh-2023-012413)
Supplement: Supplementary data [file bmjgh-2023-012413supp007.pdf]

Appendix 7: Detailed results of Genotoxicity

Genotoxicity in bidi workers

We found fifteen studies assessing the genotoxicity among the bidi workers.[1-15] There were three cross-sectional[1, 7, 12] and 12 case control studies.[2-6, 8-11, 13-15] These studies typically measured several biological markers related to toxicity(example urinary thio-ether, salivary and urinary cotinine levels) or studied genetic aberrations (example measurement of telomere, mutagenicity etc)

Urinary thio-ether levels

We found five studies assessing the urinary thio-ether levels in workers engaged in bidi rolling and tobacco processing activities.[9] [5, 6, 8, 12] *The mean urinary thioether levels in bidi workers were significantly greater than those in controls [Mean ± SE = 4.59±0.52 to 12.58 ± 2.17 µmol/mmol creatinine versus 1.83±0.34 to 6.65 ± 1.07; p<0.001].*[6, 8, 9] *Another study reported a significant elevated levels of thio- ether in the BR-S*[1] *group (4.59 + 0.52; P < 0.001) but it was lower in the BR-K*[2] *group (0.54 + 0.08; p < 0.001) compared to the control (1.83 + 0.34).*[5] *Similar results were reported by another cross-sectional study [workers working in confined (37.36±7.50) versus mixed kind of working conditions (32.78±6.20 µm) versus well-ventilated work place (18.28±4.10 µm); P<0.05] indicating that confined environment predisposes the bidi workers to inhale bidi dust and open environment tends to reduce the hazard.*[12]

Salivary and urinary cotinine levels

We found five studies evaluating the cotinine levels in bidi workers indicating high levels of tobacco dust exposure.[2, 3, 5, 6, 9]

The mean urinary cotinine levels in tobacco processors with no tobacco and tobacco habits were significantly higher than the no tobacco control group [3.46 ± 0.95 and 3.57 ± 0.46 versus 1.80 ± 0.58 mM/M creatinine; P < 0.02],[3] [poorly ventilated 0.79 ± 0.30 versus open area 0.09 ± 0.03].[5] This is indicative of increased absorption of tobacco during occupational exposure.

Other studies reported similar results with detectable levels of cotinine [(salivary = 0.84±0.26 µg/ml and urinary = 0.09±0.03 mmol/mol creatinine] in the saliva and urine of bidi workers.[2, 6, 9]

Telomere dysfunction and DNA damage

We found three studies assessing telomere length *in bidi* workers exposed to tobacco dust.[8, 10, 15] Telomere dysfunction is a marker of DNA damage with long term health consequences.

---

[1] Bidi workers who worked singly in open courtyards
[2] Bidi workers working in a poorly ventilated room

While one study reported that the average absolute telomere length/each chromosome end (kb) did not change significantly between bidi workers and control group [mean and SD = 0.75 ± 0.94 and 1.45 ± 2.76 kb; P=0.911][10], the other two studies found that the comet and tail length of telomeres showed significant increase in bidi workers occupationally exposed to tobacco dust.[8, 15]

Nicotine induced oxidative stress

We found one study evaluating the status of oxidants and antioxidants and their association with nicotine in bidi workers occupationally exposed to tobacco.[14]

The study reported a significantly high level of oxidants, lipid peroxide in the form of MDA (p<0.001) and serum nitric oxide (NO) as nitrite (p <0.001) as compared to the controls [MDA (nMol/ml) = 2.61 ± 0.25; (NO) = 55.5 ± 6.77 versus control (MDA)1.80 ± 0.48; (NO)49.14 ± 5.34; P<0.05]

There was significant decline in the levels of antioxidants, erythrocytic- Superoxide Dismutase (RBC-SOD) (P<0.001), Vitamin-C (P<0.001) and total antioxidant capacity (TAC) (P<0.001) in bidi workers as compared to the matched controls.[14]

Chromosomal aberration

We found five studies evaluated chromosomal aberration in bidi workers and tobacco processors.[4, 8, 11, 13, 15]

Bidi workers who were exposed to tobacco dust reported a significant increased chromosome aberration [mean and SD = 3.1±0.347 versus 1.4±0.15; P<0.05] as compared to the controls.[15] Another study which was conducted on tobacco user bidi workers and non-user bidi workers reported similar results [mean and SD (non-user bidi workers 1.4 ± 0.25); P<0.0002 versus control 0.04 ± 0.04] [mean and SD (tobacco user bidi workers 1.97 ± 0.54); P<0.05 versus control 0.82 ± 0.28] when compared with non-tobacco user controls.[4] Others studies also reported bidi workers exposed to bidi tobacco dust significantly increased (P < 0.05) chromosome aberration in all age groups than that of age matched controls [8, 11, 13] indicating that occupational exposure to tobacco leads to a considerable genotoxicity among tobacco processors/ bidi workers.

Salivary sialic acid levels a pre-disposing factor of oral cancer

We found one study assessing the levels of sialic acid in bidi workers.[1]

The study reported high salivary sialic acid level of bidi workers [mean and SD (mg/dL) =3.26 ± 5.00; 3.06 ± 6.0) as compared to no tobacco exposure. However, it was statistically non-significant.

Serum sialic acid is a sensitive tumour marker and increased levels might cause oral cancer.

Metal toxicity

We found one study assessing occupational exposure to toxic metals using fingernails of the bidi workers.[7]

The study reported higher levels of lead (44.2 µg g^{-1}) in the fingernails of bidi workers as compared to other metals. In bidi workers Cd (3.86 µg g^{-1}) and Cr (2.72 µg g^{-1}) were particularly higher than the normal level (0.25 µg g^{-1}) of control subject not exposed in occupational working environment. Exposure to heavy metals could affect normal bodily function and could lead to chromium toxicity causing renal damage and increase cancer risk.[7]

Mutagenicity

We found two studies assessing urinary mutagenicity due to tobacco exposure in bidi workers with no tobacco habit and tobacco processors (no tobacco habit and masheri habit).[3, 6]

The study reported exposure to tobacco during bidi rolling resulted in an increased mutagenicity in TA98 in absence of metabolic activation, but no mutagenic activity was detected in TA100. This implies genotoxic hazard of occupational exposure to tobacco.[6]

The majority of the urine samples from no tobacco habit and control were non-mutagenic in the presence or absence of S9 while those from masheri habit and controls were mutagenic to TA98 and TA102 strains upon metabolic activation.[3] This is indicative of exposure to occupational geno-toxicants causing DNA damage and high risk of causing cancer.

Twelve of 15 studies were conducted on female bidi workers. [4-6, 8, 10, 12, 15],[2, 3, 7, 9, 11] The bidi workers were employed in either bidi establishment (4 studies)[3, 4, 7, 11] or at OAME (1 study).[5]

1. Ancy, R.J., et al., *Comparative evaluation of salivary sialic acid levels among beedi rollers and tobacco users in Mangalore, South India.* Cureus, 2021. **13**(7): p. e16651.
2. Bagwe, A.N. and R.A. Bhisey, *Occupational exposure to tobacco and resultant genotoxicity in bidi industry workers.* Mutat Res, 1993. **299**(2): p. 103-109.
3. Bagwe, A.N. and R.A. Bhisey, *Occupational exposure to unburnt bidi tobacco elevates mutagenic burden among tobacco processors.* Carcinogenesis, 1995. **16**(5): p. 1095-1099.
4. Bhisey, R.A., et al., *Biological monitoring of bidi industry workers occupationally exposed to tobacco.* Toxicol Lett, 1999. **108**(2-3): p. 259-65.
5. Bhisey, R.A. and R.B. Govekar, *Biological monitoring of bidi rollers with respect to genotoxic hazards of occupational tobacco exposure.* Mutat Res, 1991. **261**(2): p. 139-47.
6. Bhisey, R.A., R.B. Govekar, and A.N. Bagwe. *Toxic effects of exposure to tobacco among bidi rollers*. in *Control of tobacco- related cancers and other diseases International Symposium*. 1992.

7. Moses, M.F. and J.J. Prabakaran, *Evaluation of occupational exposure to toxic metals using fingernails as biological indicators.* Res J Environ Toxicol, 2011. **5**(1): p. 65-70.
8. Gautam, D., A. Khanna, and R. Bhandari, *Genotoxic effects of tobacco dust exposure on bidi rollers.* Int. J. Pharm. Med. Biol. Sci., 2015. **4**(1): p. 20-23.
9. Govekar, R.B. and R.A. Bhisey, *Elevated urinary thioether excretion among bidi rollers exposed occupationally to processed tobacco.* Int Arch Occup Environ Health, 1992. **64**(2): p. 101-4.
10. Kanipakam, Y., Nagaraja V, Rajaram S, et al. and 2021, *Association between occupational exposure to tobacco dust and absolute telomere length: A cross-sectional study on female beedi workers.* World J Dent, 2021. **12**(5): p. 417–422.
11. Mahimkar, M.B. and R.A. Bhisey, *Occupational exposure to bidi tobacco increases chromosomal aberrations in tobacco processors.* Mutat Res, 1995. **334**(2): p. 139-44.
12. Shukla, P., A. Khanna, and S.K. Jain, *Working condition: A key factor in increasing occupational hazard among bidi rollers: A population health research with respect to DNA damage.* Indian J Occup Environ Med, 2011. **15**(3): p. 139-41.
13. Sundaramoorthy, R., et al., *Clinical, cytogenetic and CYP1A1 exon-1 gene mutation analysis of beedi workers in Vellore region, Tamil Nadu.* Asian Pac J Cancer Prev, 2013. **14**(12): p. 7555-60.
14. Swami, S., et al., *Absorption of nicotine induces oxidative stress among bidi workers.* Indian J Public Health, 2006. **50**(4): p. 231-5.
15. Khanna, A., et al., *Tobacco dust induced genotoxicity as an occupational hazard in workers of bidi making cottage industry of central India.* Toxicology International, 2014. **21**(1): p. 18-23.
